# Stratodynamics: A New Theory of Layered Biological Response

**DOI:** 10.3390/biology15161426

**Published:** 2026-08-18

**Authors:** Burim N. Ametaj

**Affiliations:** Department of Agricultural, Food and Nutritional Science, University of Alberta, Edmonton, AB T6G 2P5, Canada; burim.ametaj@ualberta.ca

**Keywords:** stratodynamics, homeostasis, allostasis, biological response, mismatch reduction, layered architecture, inter-layer coupling, chronic disease, multiscale physiology, vertebrate physiology

## Abstract

Physiology has long explained organismal stability through homeostasis, allostasis, feedback regulation, and related systems concepts. These frameworks remain essential, but each is most precise at a particular scale or mode of response. This manuscript proposes stratodynamics as a new theory of layered biological response in vertebrate physiology. The framework holds that biological response is organized through seven functional layers: molecular, subcellular, cellular, tissue, organ, systemic, and organismal. The layer names denote the compositional scale of the focal response unit rather than fixed anatomical compartments; layer assignment is functional and response-specific. Each layer reduces its own form of mismatch between actual and required state through a scale-appropriate architecture: molecular-state transition, intracellular integration, cellular-state transition, tissue program, organ output, systemic feedback, or organismal anticipation. The layers are coupled upward, downward, and laterally, allowing local events to become systemic responses and allowing organismal state to shape lower-layer readiness. Stratodynamics locates rather than replaces previous theories: homeostasis is preserved as the canonical systemic architecture, allostasis as organismal predictive regulation, and tissue repair, resolution biology, bioelectricity, and thermodynamic self-organization as complementary layer-specific contributors. The framework is presented as a theoretical synthesis and research program for veterinary and medical sciences.

## Simple Summary

Living organisms survive because their molecules, cells, tissues, organs, body systems, and whole-body behavior respond together when conditions change. Classical homeostasis explains how the body keeps important variables such as temperature, blood pressure, glucose, pH, and calcium within safe ranges, but many biological responses are broader than simple return to a set point. Immune defense, wound healing, lactation, stress adaptation, growth, and chronic disease involve many levels of organization acting at the same time. This manuscript proposes stratodynamics as a new theory of layered biological response in vertebrates. The central idea is that each biological layer reduces its own kind of mismatch between the current state and the state required for continued function. Health reflects coherence among layers, whereas disease may persist when this coherence is not restored. This framework may help veterinary and medical sciences understand complex diseases that cannot be explained by one molecule, cell type, organ, or systemic variable alone.

## 1. Introduction: The Constancy Bernard Saw

The central question of modern physiology begins with Claude Bernard. In *Introduction à l’étude de la médecine expérimentale* (An Introduction to the Study of Experimental Medicine) (1865) [1], Bernard established the concept of the milieu intérieur, the internal environment within which the cells of a complex organism actually live, as one of the foundational objects of experimental medicine. Thirteen years later, in the lectures collected as *Leçons sur les phénomènes de la vie communs aux animaux et aux végétaux* (Lectures on the Phenomena of Life Common to Animals and Plants) (1878) [2], he gave this recognition its most famous formulation:

“La fixité du milieu intérieur est la condition de la vie libre et indépendante [2].”

“The fixity of the internal environment is the condition of free and independent life.”

A complex animal does not expose its cells directly to the external world. An internal medium of extracellular fluids, electrolytes, gases, nutrients, temperature, pH, osmotic forces, hormones, immune mediators, and metabolic substrates is maintained within viable limits despite heat, cold, fasting, exercise, infection, pregnancy, lactation, injury, and stress.

Bernard identified the condition of physiological freedom but left its mechanism unresolved: how is internal constancy achieved, through which architectures, and at what scales?

The first systematic answer came from Walter B. Cannon. In 1926, Cannon introduced the term homeostasis [3,4,5], and in The Wisdom of the Body (1932) he developed it into a comprehensive framework for physiological regulation [6,7]. Cannon’s contribution was decisive: regulated variables are defended within tolerable ranges through sensors, integrators, effectors, and negative feedback. He thereby transformed Bernard’s recognition of internal constancy into a mechanistic architecture.

An immune response does not simply return cells to baseline; it produces clonal expansion, effector differentiation, antibody production, memory, and sometimes tolerance. A wound does not defend a setpoint; it initiates hemostasis, inflammation, proliferation, remodeling, and, in some organisms, regeneration. An embryo does not maintain a pre-existing condition; it constructs tissues and organs that did not previously exist. A learning nervous system does not preserve its synaptic architecture; it modifies it and retains the modification. A lactating mammary gland does not merely restore an earlier state; it reorganizes metabolism, epithelial secretion, vascular supply, endocrine sensitivity, and immune defense to meet a new physiological demand. These processes are not failures of homeostasis. They are biological responses of a different kind.

Subsequent frameworks extended this account: Selye described temporally organized adaptation; cybernetics formalized feedback and control; non-equilibrium thermodynamics explained maintenance of living order; allostasis introduced anticipatory regulation; resolution biology and bioelectricity defined active tissue programs; and the free energy principle formalized predictive self-maintenance [8,9,10,11,12,13,14,15,16,17,18,19,20,21,22,23]. Work on causal emergence further supported the explanatory relevance of higher organizational levels [24,25,26]. Together, these accounts show that biological response includes correction, prediction, construction, repair, and self-organization.

These frameworks capture genuine but partial modes of biological response. Homeostasis is most precise for defended systemic variables; allostasis for organismal prediction; thermodynamics for the physical maintenance of living order; and resolution biology and bioelectricity for tissue programs. Their limits point not to competition, but to a larger architecture in which distinct response modes operate at different scales and are coupled into one organismal response.

This paper proposes the term stratodynamics for this layered architecture. The combining form strato- denotes layer or stratum; dynamics, from Greek dynamis, denotes force, power, or capacity to act. Stratodynamics therefore names not a static layered condition, but a layered dynamics of response. The term is constructed in deliberate continuity with homeostasis and allostasis. Homeostasis names stability through defended similarity; allostasis names stability through predictive change; stratodynamics names the layered coherence through which multiple forms of biological response are coordinated across scales.

Stratodynamics provisionally organizes vertebrate response into seven functional layers: molecular, subcellular, cellular, tissue, organ, systemic, and organismal. Each reduces a layer-specific mismatch through a characteristic architecture: molecular transition, intracellular integration, cellular-state transition, tissue program, organ output, systemic feedback, or organismal anticipation.

The unifying principle is that life persists by reducing mismatch at each scale through layer-appropriate architectures. “Required” denotes a state compatible with continued function under prevailing conditions, not conscious purpose or fixed design; its meaning therefore changes across layers.

The layers are coupled upward, downward, and laterally. Lower-layer events contribute to higher-layer integration; organismal and systemic context shape lower-layer readiness; and components within a layer coordinate one another. Response is therefore vertical across scales, horizontal within scales, and temporal across their different rates of action.

Section 2 traces the conceptual lineage; Section 3, Section 4 and Section 5 define stratodynamics, mismatch reduction, and its hierarchical foundations; Section 6 and Section 7 develop the layers and their couplings; Section 8 examines veterinary and medical applications; and Section 9 and Section 10 state limitations, predictions, and conclusions.

This article is a theoretical synthesis, not a systematic review. It focuses on historical frameworks and contemporary theories that most directly overlap with stratodynamics in multiscale agency, whole-body integration, viability, critical transitions, or predictive self-maintenance. Its purpose is to state a testable architecture rather than exhaust the specialized literatures.

Vertebrate challenges recruit several organizational scales simultaneously. Lactation, infection, sepsis, exercise, and wound repair can each involve receptor engagement, intracellular signaling, cell activation, tissue remodeling, organ reprogramming, systemic deviation, and organismal behavior. Stratodynamics is proposed to keep these descriptions connected rather than allowing one scale to replace the others.

In veterinary medicine, mastitis, lameness, and transition-cow disorders illustrate this coupled complexity; comparable multiscale interactions occur in human chronic disease.

The present paper therefore focuses on complex multicellular animals, especially vertebrates. The framework may later be adapted to plants, microbes, embryos, colonies, ecosystems, or supraorganismal systems, but those applications require domain-specific changes. Plants, for example, have distributed regulation without a nervous system; microbes have population-level and ecological forms of response without organs; colonies and ecosystems have organizational scales beyond the individual organism. These domains are important, but they are not the main subject of the present article. The aim here is to state a vertebrate-centered theory clearly enough that it can be tested, criticized, and extended.

**Figure 1:**
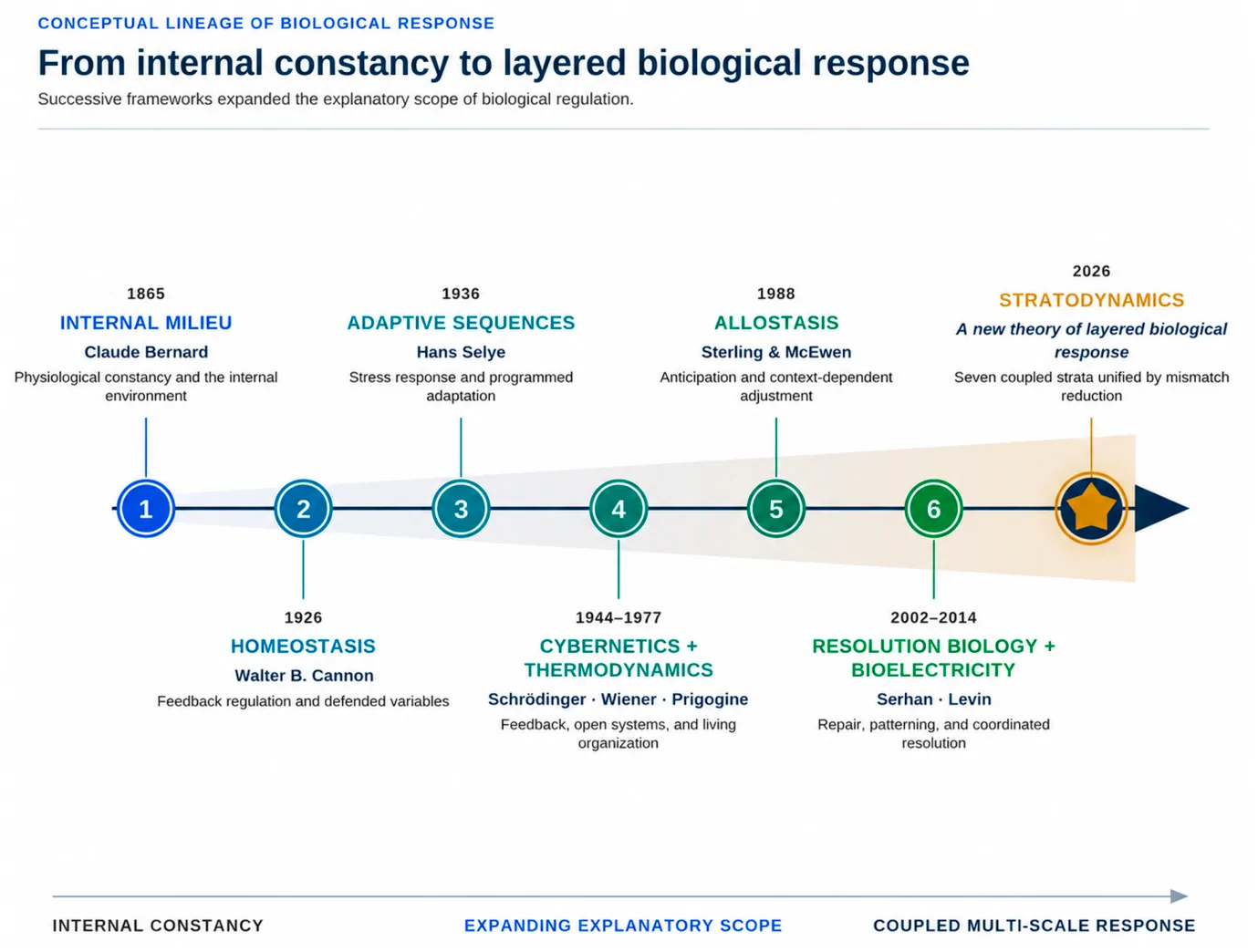
From internal constancy to layered biological response. Claude Bernard identified the problem of the internal environment; Cannon formalized systemic feedback as homeostasis; Selye emphasized adaptive sequences; cybernetics and thermodynamics supplied general principles of regulation and living order; allostasis introduced predictive adjustment; and modern work on resolution biology and bioelectricity revealed tissue-level regulatory programs. Stratodynamics integrates these partial frameworks into a new theory of layered biological response. Reading the figure: the horizontal axis is historical time and the numbered nodes mark successive frameworks, each labeled with its year, originator, and central contribution; the widening band indicates the progressive broadening of explanatory scope, from constancy of a single internal milieu to coupled response across scales. The final node is not a further predecessor but the synthesis proposed here. The color progression reflects the expanding scope of biological regulation: blue represents early concepts of internal constancy and homeostasis; teal marks the transition toward adaptive and systems-level regulation; green represents modern concepts of predictive, tissue-level, and multiscale regulation; and gold highlights Stratodynamics as the integrative synthesis of these preceding frameworks. The widening gray-to-gold band reinforces this progressive expansion in explanatory scope.

This historical progression is summarized in Figure 1.

## 2. The Lineage: A Genealogy of Partial Answers

The 160 years since Bernard’s milieu intérieur can be read as a sequence of partial answers to one question: how is the internal viability required for free and independent life maintained? Physiology, cybernetics, thermodynamics, immunology, developmental biology, and systems theory each identified a genuine architecture or constraint. This section locates the principal contributions at the scales where they are most precise and shows how they motivate a layered synthesis.

### 2.1. Bernard: The Recognition of the Internal Environment

Bernard’s contribution was not a complete theory of regulation. It was a foundational recognition: complex life is not lived directly in the external world. Between the cells of a complex organism and the environment through which the organism moves lies an internal environment whose physical and chemical properties are protected from external variation. The cells of a vertebrate experience a medium whose temperature, osmolarity, ionic composition, oxygen tension, glucose concentration, and pH are maintained within viable limits even while the organism encounters heat, cold, fasting, exertion, infection, injury, and environmental change.

The phrase milieu intérieur gave physiology its central object [1,27,28]. Every later framework of biological regulation, homeostasis, allostasis, cybernetic control, thermodynamic self-organization, active inference, and stratodynamics, can be understood as an attempt to answer the question Bernard left open: how is the constancy of the internal environment achieved?

What Bernard did not address, and what the concept of milieu intérieur alone cannot address, is the scale-dependence of biological regulation. Bernard wrote at the scale of the organism and its fluids: blood, lymph, and tissue fluid. He did not distinguish the constancy of a systemic variable such as blood pH from the stability of a cellular state, the organization of a tissue, the output of an organ, or the predictive adjustment of the organism as a whole. His framework is foundational because it operates above these distinctions. It is limited for the same reason.

### 2.2. Cannon: The Architecture of Homeostasis

If Bernard recognized the fact of internal regulation, Cannon specified one of its major architectures. Writing two generations after Bernard, with a mature experimental physiology available to him, Cannon proposed that regulated variables of the internal environment are maintained within ranges by specific control systems. He introduced the term homeostasis in 1926 and elaborated the framework in his 1929 paper in Physiological Reviews and in The Wisdom of the Body (1932) [4,5,6].

The architecture is the familiar sensor–integrator–effector triad. Sensors detect the value of a regulated variable, and effectors are engaged as that value crosses thresholds built into the sensing machinery, so that the variable settles within a defended range. Negative feedback closes the loop. This architecture recurs throughout physiology: baroreceptors defend arterial pressure; chemoreceptors contribute to regulation of blood gases and pH; thermoregulatory circuits defend body temperature; pancreatic islets regulate blood glucose; the parathyroid axis defends plasma calcium; renal and hypothalamic systems defend osmolarity and volume.

Yet Cannon’s framework is not universal. It describes the regulation of systemic variables most precisely. It does not describe molecular-state transitions, where no setpoint is defended; intracellular signaling cascades, where signals are integrated through thresholds, oscillations, and network dynamics; cellular-state transitions, where cells may adopt new phenotypes rather than return to baseline; tissue programs, where wounds heal through temporal phases rather than simple correction; or organismal anticipation, where the target of regulation shifts before deviation occurs. Cannon’s framework is correct at the scale for which it was designed. It becomes incomplete only when treated as the general architecture of all biological response.

### 2.3. Selye: Programmed Adaptive Sequence

Selye’s contribution introduced a mode of response that Cannon’s framework cannot fully describe. In his 1936 letter to Nature, and later in The Stress of Life (1956) and related work, Selye observed that diverse stressors, cold, trauma, infection, toxins, and psychological strain, elicit a common sequence of physiological changes in the organism [8,9]. He named this sequence the general adaptation syndrome and described three phases: alarm reaction, resistance, and exhaustion.

The conceptual importance of Selye’s framework is that it identifies biological response as temporal and programmed rather than merely corrective. The general adaptation syndrome is not a feedback loop defending one variable around a setpoint. It is a trajectory through physiological state space. It has phases. It has duration. It has possible outcomes: adaptation, recovery, exhaustion, or death. Selye therefore introduced the idea that the body has response programs as well as defended variables.

Selye also came closer to a layered view than is often appreciated. He distinguished systemic general adaptation from local adaptation around injured tissues. The Local Adaptation Syndrome recognized that a local tissue injury generates a programmed inflammatory response, while systemic stress alters local tissue reactivity and local injury feeds back onto the systemic organism. In retrospect, this was an early recognition of tissue–system coupling. What Selye did not do was generalize programmed sequence into a multi-layer architecture of biological response. His contribution was the recognition of adaptive sequence and local–systemic coupling; the layered synthesis came later [10].

### 2.4. Cybernetics: Mathematical Language for Regulation

Wiener and Ashby gave regulation a mathematical language of feedback, control, stability, communication, state space, and requisite variety [11,12]. Cybernetics clarified how organized behavior persists under disturbance and supplied physiology with a general formal vocabulary. It did not, however, specify how molecules, organelles, cells, tissues, organs, systemic networks, and whole organisms generate regulatory variety through different biological mechanisms. Stratodynamics retains the cybernetic language while making its scale-specific biological implementation explicit.

### 2.5. Schrödinger and Prigogine: The Thermodynamic Ground

In this broad thermodynamic sense, living organisms belong to the class of far-from-equilibrium systems. Their order is not an equilibrium structure but a continuously maintained process. Yet an organism is not merely a whirlpool with chemistry added. It is a regulated dissipative structure in which metabolism, signaling, development, repair, prediction, and multi-scale control are built upon the thermodynamic substrate. Prigogine’s work, developed with Nicolis and others and synthesized in Self-Organization in Nonequilibrium Systems (1977), established the physical ground of biological self-organization.

Stratodynamics therefore builds directly on the thermodynamic tradition while extending it. It accepts that every layer of biological regulation must be paid for by energy throughput and entropy export. But it adds that each scale uses a distinct biological architecture to build, regulate, repair, and coordinate its order. Thermodynamics explains why biological order is possible. Stratodynamics asks how that order is organized across layers.

In the present article, these physical concepts are used in a simple biological sense. A living organism is not a static object; it is a maintained process. Its order persists because nutrients, oxygen, water, ions, heat, waste products, and information continuously move through it. The physical language of energy flow and entropy export underscores that biological organization has a cost. However, it does not replace physiology. It explains why living order must be continuously maintained; it does not specify how hemoglobin changes state, how mitochondria signal stress, how macrophages polarize, how tissues resolve inflammation, how organs alter output, or how animals anticipate future demand.

The same simplifying approach is taken for mathematical concepts. Feedback is used here in the physiological sense of sensors, effectors, and regulated variables. Requisite variety means only that a regulatory system must have enough response options to deal with the disturbances it encounters. The present theory does not require causal emergence, non-additivity, or the claim that the whole is more than the sum of its parts. Fever, tissue repair, organ output, locomotor behavior, and lactational state are treated as measurable responses assigned to particular compositional scales, not as evidence for an emergent ontology.

### 2.6. Sterling, Eyer, and McEwen: Predictive Regulation and Allostatic Load

Sterling and Eyer, in their 1988 chapter “Allostasis: A New Paradigm to Explain Arousal Pathology,” introduced one of the most important revisions to Cannon’s framework, and McEwen later developed the central role of the brain in stress adaptation and allostatic load [16,29]. Their observation was empirical and decisive: many important regulated variables are not defended near constant values. Arterial pressure, cortisol, glucose, body temperature, immune tone, and metabolic state vary systematically with circadian phase, anticipated demand, life stage, and environmental context [15]. Cortisol rises before waking. Blood pressure rises before exertion. Insulin secretion can begin at the sight, smell, or expectation of food, before absorbed glucose enters the circulation. In these cases, the organism is not merely correcting deviation after it occurs. It is anticipating demand and pre-adjusting physiology in advance.

The contribution of allostasis is profound. It establishes that whole-organism regulation is not only reactive but predictive. The target of regulation is not always a fixed value; it can be a context-dependent target generated by the organism’s anticipation of future demand. This recognition changes how Bernard’s milieu intérieur should be understood. Its constancy is not maintained only by correcting deviations. It is also maintained by predicting which internal states will soon be required and moving toward them in advance.

### 2.7. Serhan: Active Resolution of Inflammation

Serhan and colleagues showed that inflammation terminates through an active tissue program rather than passive disappearance of inflammatory signals [17,18,30]. Specialized pro-resolving mediators limit neutrophil recruitment, promote efferocytosis and macrophage-state transitions, enhance clearance, and permit restoration of tissue function. Resolution biology therefore demonstrates that a response must not only begin appropriately but also complete its sequence. Chronic inflammation may reflect failure to enter or complete resolution and repair, a tissue-level program-completion failure rather than simply excessive initiation.

### 2.8. Levin: Bioelectric Organization of Tissues

Levin and colleagues identified bioelectric patterning as a measurable, causally active dimension of tissue organization [19,20]. Collective voltage gradients, ionic currents, gap-junctional communication, and wound electric fields influence morphogenesis, regeneration, and cell migration [31,32,33]. Individual cells possess membrane potentials, but tissues generate coordinated electrical patterns no single cell contains alone. This provides a strong empirical basis for distinguishing tissue-level organization from cellular response while preserving their coupling.

### 2.9. Friston: Free Energy, Prediction, and the Limits of Universal Formalism

The free-energy principle is one of the most explicit formal accounts of self-maintenance and organism–environment coupling [21,22,23,34], but stratodynamics does not depend on it. Its scope remains debated [35,36], cognitive language can be misleading outside nervous systems, and variational free energy is not the Gibbs free energy or metabolic energy measured in physiology. The framework is therefore located where it is strongest—organismal prediction and action—while stratodynamics asks how self-maintenance is implemented through molecular, cellular, tissue, organ, and systemic mechanisms.

### 2.10. Emergence and Higher-Level Explanation: A Boundary of the Present Theory

Integrated-information and causal-emergence theories ask whether system-level organization possesses explanatory or causal properties not captured by isolated components [24,25,26]. Stratodynamics does not adopt that proposition. Its upward, downward, and lateral couplings are operational dependencies among prespecified response units and can be tested without assuming irreducibility, non-additivity, or emergence. These theories are therefore included only to delimit the scope of stratodynamics, not as foundations of it.

### 2.11. Davies: Adaptive Homeostasis and the Modulation of Regulatory Capacity

Davies’ adaptive homeostasis describes transient, signal-driven modulation of protective capacity by sub-toxic, non-damaging cues [37]. In the Keap1–Nrf2 example, low hydrogen peroxide can expand antioxidant and proteostatic capacity, which contracts after the signal is removed [38]. This differs from hormesis, a biphasic dose–response relation [39], and illustrates how cellular protective capacity itself can be adjusted. Apparent remission may therefore not equal removal of the underlying disease mechanism [40].

### 2.12. Synthesis of the Lineage

Stratodynamics names this layered architecture. It is offered not as a rejection of the lineage, but as one synthesis of it: a framework that gives each predecessor its proper scale and shows how the scales fit together. The remainder of the paper develops this framework formally. For readers approaching Bernard through English, the later translation of his lectures remains a useful bridge between the original French formulation and modern physiological language [41].

## 3. The Concept of Stratodynamics

The term is defined formally as follows:

Stratodynamics is the principle that biological response to a stimulus is organized in scale-specific layers, each operating through its own architecture, timescale, and regulatory logic. The coherent response of a living organism is the integrated activity of these layers, each addressing its own form of mismatch between actual and required state. No single architecture, neither homeostatic feedback, nor allostatic prediction, nor developmental construction, nor tissue repair, nor thermodynamic self-organization, describes biological response in general. Each is correct at its proper scale. Together they constitute stratodynamics: the layered coherence of the living response. Plain-language definitions of recurring terms used in stratodynamics are provided in Box 1.

Box 1 **Plain-language definitions used in stratodynamics and glossary of recurring terms.**

**Homeostasis** refers to feedback defense of systemic variables, such as pH, osmolarity, glucose, arterial pressure, oxygenation, calcium, or temperature, within viable ranges.

**Allostasis** refers to predictive adjustment before or during expected demand.

**Mismatch** refers to the difference between the present state of a biological layer and the state required for continued function under current or anticipated conditions.

**Coupling** refers to communication and constraint among layers: upward from molecular and cellular events toward organismal response, downward from organismal and systemic state toward molecular readiness, and laterally among components within the same layer.

**Layered coherence** refers to the coordinated activation of biological layers with appropriate timing, magnitude, localization, and termination. Disease may begin at one layer, but it often persists when layered coherence is not restored.

**Response unit.** the focal biological entity whose response is being analyzed—a molecule, signaling module, cell, tissue region, organ, regulated variable, or the whole organism. Layer assignment applies to the response unit, not to the layer as an undifferentiated whole.

**Viable set.** the set of states compatible with continued function of a response unit under prevailing conditions, specified independently of the response being studied.

**Required state.** shorthand for membership in the viable set; it implies no purpose, design, or foresight.

**Corrective reduction.** return of a variable toward a previously maintained viable range.

**Adaptive reduction.** transition toward a new viable state rather than restoration of the prior one.

**Coupling dysfunction.** attenuation, exaggeration, mistiming, reversal, or ectopic activation of an expected dependency, judged against an independently specified reference dependency.

**Stratum.** used interchangeably with layer for a scale of response satisfying the criteria in Section 5.3.

The seven-layer scheme is a provisional functional architecture for complex multicellular animals, not a universal law. Layers identify the compositional scale of the focal response unit and are assigned by response architecture, not rigid anatomical compartments. Other biological domains may require modified partitions. The claim is therefore not that every system has exactly seven levels, but that vertebrate response is scale-specific, coupled, and empirically partitionable.

**Figure 2:**
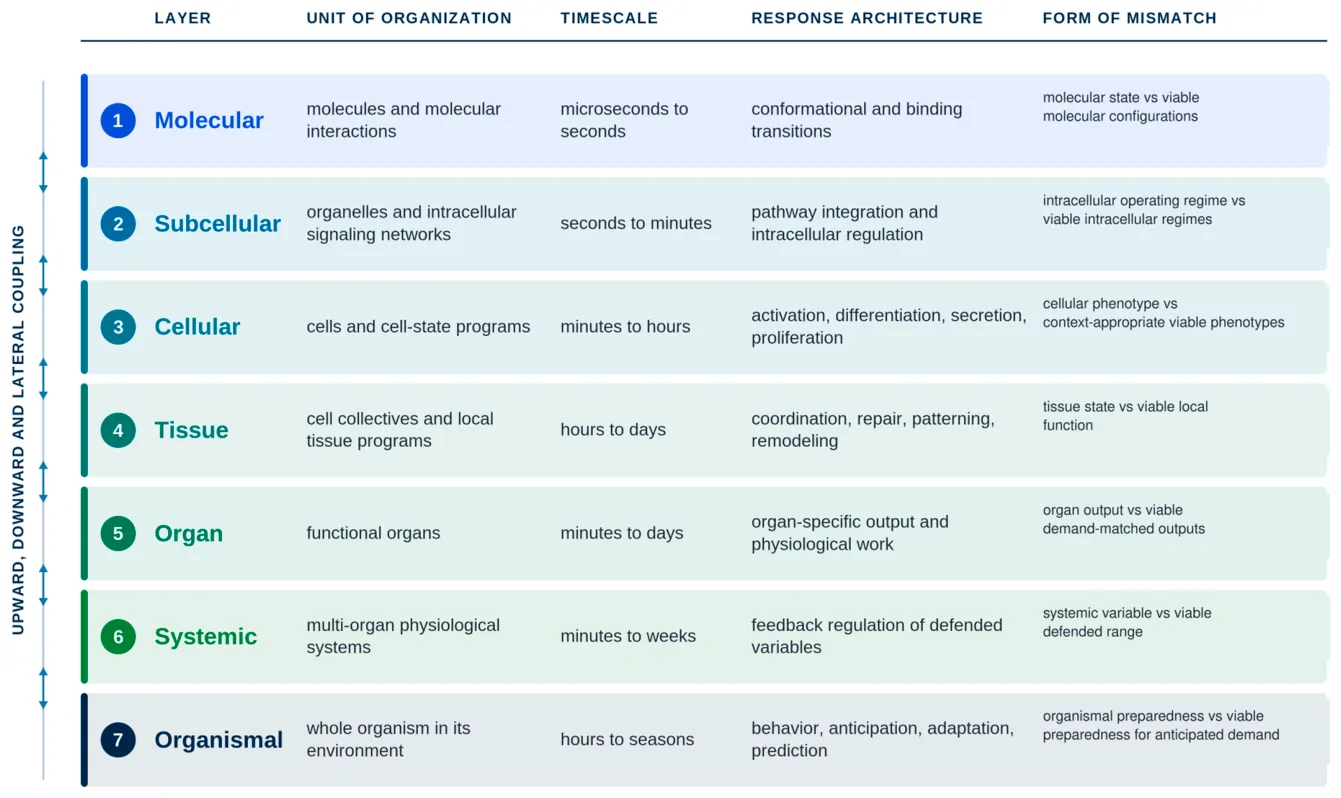
The seven functional layers of stratodynamics. Biological response in vertebrates is organized across molecular, subcellular, cellular, tissue, organ, systemic, and organismal layers. Each layer has its own unit of organization, characteristic timescale, response architecture, and form of mismatch between the observed state and an independently specified viable set. The layers are distinct but coupled into one coherent biological response. Reading the figure: each row is one stratum, identified by number and name and read left to right as its unit of organization, characteristic timescale, response architecture, and the form of mismatch it reduces. Colors distinguish strata and are used consistently throughout the figures. Timescales are broad operating ranges that overlap substantially and are neither sharp boundaries nor a ranking of layers. The double-headed arrows at the left indicate that adjacent strata are coupled in both directions and laterally within each stratum.

The seven functional layers of stratodynamics are summarized in Figure 2.

Stratodynamics makes three central claims:

**Claim 1. Layer-Specific Architecture**

The first claim is that each layer of biological organization has its own characteristic architecture of response, and that no single regulatory architecture applies across all biological scales.

Cannon’s sensor–integrator–effector triad is the architecture of classical systemic homeostasis. It is correct for systemic variables such as blood pH, plasma osmolarity, arterial pressure, blood glucose, body temperature, blood gases, and plasma calcium. It is not, however, the architecture of molecular ligand binding, mitochondrial threshold activation, cellular-state transition, tissue repair, organ output, or whole-organism anticipation. These processes do not simply repeat the same regulatory design at different sizes. They operate through different mechanisms because they address different kinds of biological mismatch.

**Claim 2. Inter-Layer Coupling**

Inter-layer coupling is the second claim: measurable upward, downward, and lateral dependencies integrate the strata into one organismal response (developed in Section 7).

**Claim 3. Coupling Vulnerability in Disease**

The third claim is that many diseases of complex multicellular organisms, especially chronic, multifactorial, relapsing, and poorly resolving diseases, preferentially involve failures of coupling among layers. This claim is proposed as a testable hypothesis generated by the framework, not as an established universal law.

Some diseases clearly originate primarily within one layer. Monogenic enzymopathies, ion-channel disorders, mitochondrial DNA mutations, protein misfolding diseases, chromosomal abnormalities, acute toxic injury, traumatic damage, nutritional deficiencies, and certain infectious lesions may begin as relatively localized failures at the molecular, subcellular, cellular, or tissue scale. Stratodynamics does not deny such origins. Rather, it asks how such local failures propagate upward and downward, and why some become stabilized as systemic or organismal disease.

Chronic multifactorial disease may persist when inter-layer coupling fails to restore coherence. Candidate examples include failed resolution of inflammation, mismatches among metabolic prediction and systemic regulation, propagation of altered immune tolerance into tissue injury, and self-reinforcing interactions among cellular activation, matrix mechanics, inflammation, and organ dysfunction. These are testable framework-generated hypotheses, not established universal mechanisms.

The clinical consequence, if this claim holds, is a new diagnostic logic. The question is not only which molecule, cell type, organ, or systemic variable is abnormal. The question is also where the originating mismatch occurred, how it propagated across layers, and which coupling now sustains the pathological state. The therapeutic consequence is equally important: durable treatment may require restoring inter-layer coherence rather than suppressing only the most visible downstream marker. In chronic disease, the target may be a failed transition, a mistimed signal, a distorted scaling relationship, or an unresolved coupling between layers.

This claim must be tested. Its validation would require layer-resolved longitudinal studies, multi-scale measurements, causal modeling, and interventions directed at proposed originating layers or failed couplings. The framework’s present contribution is to make these questions precise enough to be investigated.

The contribution of stratodynamics is architectural and synthetic: biological response is layered; each layer has a characteristic architecture; the layers are coupled; and persistent disease may reflect failure to restore coherence. This organization supplies three consequences developed below: a common mismatch-reduction principle, a diagnostic and therapeutic research logic focused on failed coupling, and a pedagogical framework for organizing physiology from molecules to whole-organism behavior.

## 4. The Unifying Principle: Biological Response as Mismatch Reduction

A taxonomy can name seven layers, but a theory must identify what their different architectures accomplish in common. Stratodynamics proposes that shared logic as mismatch reduction.

**The proposed unifying principle is this:**

Biological response, at every scale, is the reduction of mismatch between actual and required state, where the meaning of “required” varies systematically with scale and the architecture of response varies accordingly.

The term “required” does not imply conscious purpose, design, or teleology. It refers to the state compatible with continued function at that layer under prevailing conditions. At one layer, “required” may mean the thermodynamically appropriate molecular configuration. At another, it may mean an intracellular signaling state below or above a defined activation threshold. At another, it may mean the cellular phenotype appropriate to context. At another, it may mean restoration of tissue integrity. At the systemic layer, it may mean return of a variable to its defended range. At the organismal layer, it may mean preparedness for anticipated future demand.

To serve as the unifying principle of a theory rather than as a description, these terms are defined precisely. A **challenge** is any internal or external perturbation that alters, or is projected to alter, the state of one or more response units. For each focal biological variable, response unit, or state vector at layer *i*, a **viable set** V*_i_*(t) is specified independently of the observed response. This viable set comprises the states compatible with continued function of the relevant unit under prevailing conditions. The state variable *X_i_*(t) denotes the state of that focal response unit or state vector, not the state of the layer as a whole. A **mismatch** is a discrepancy between the current or projected *X_i_*(t) and its independently specified viable set V*_i_*(t). It is not defined relative to a single “required” value, since biological variables are generally maintained within viable ranges rather than at unique setpoints, and specifying the viable set independently of the response prevents the definition from becoming circular. Throughout the notation, *i* indexes a focal response unit, biological variable, or state vector assigned to one of the seven layers; it does not represent the layer as an undifferentiated whole.

Actual or projected mismatch is the discrepancy toward whose reduction a response is directed. In reactive responses, detection or internal representation of an existing mismatch initiates or sustains the response; in anticipatory responses, a detected challenge serves as a cue for a likely future mismatch before that mismatch is fully expressed. In anticipatory applications, the projected state and the corresponding viable set must be defined over the same stated prediction horizon. Mismatch reduction is not the inevitable outcome of every response but the criterion by which successful correction or adaptation is identified. It is local and context-dependent, and it may fail: a response may reduce one mismatch while generating another, may move the system to a new viable state, or may not succeed at all. Persistent or propagating mismatch is proposed as a central contributor to chronic disease.

Two modes of mismatch reduction are distinguished because they represent different dynamical outcomes that may occur within and across layer architectures. In **corrective reduction**, the response returns a variable toward a previously maintained viable range —the mode characteristic of regulated-range defense. In **adaptive reduction**, the response drives a transition toward a new viable state rather than restoring the prior one—the mode characteristic of developmental and reparative responses, as well as some anticipatory responses. The distinction is between restoration and transition, not between two kinds of discrepancy. These modes are analytically distinct but not mutually exclusive: a single response may restore one regulated variable while shifting another toward a new viable state.

Formally, the mismatch associated with focal response unit *i* may be written

(1) $$M_{i}(t)=d[X_{i}(t),\;V_{i}(t)]$$

Here Xi(t) is the observed or projected state, Vi(t) the independently specified viable set, and d[·,Vi] a system-appropriate distance that vanishes within the viable range. Equation (1) is a scaffold for system-specific models, not a universal fitted law.

Mismatch is related to, but broader than, the control-theoretic concept of error. Error usually denotes the signed difference between an observed value and a specified reference value, whereas stratodynamic mismatch denotes the distance between an actual or projected state and an independently specified viable set. Mismatch therefore does not necessarily indicate malfunction; it may arise during normal correction, adaptation, development, repair, or anticipation.

**The framework can be stated in one sentence:**

Life persists not by maintaining constancy at one scale, but by actively reducing mismatch at every scale through layer-appropriate architectures. A worked example applying these definitions to body-temperature regulation is provided in Box 2.

Box 2 **A worked example of the definitions: body-temperature regulation.**

To make the definitions concrete, consider body temperature under cold exposure and infection. The example is familiar and measurable, but the same procedure can be applied at other scales by specifying the response unit, state variable, viable set, and distance measure.

**Focal response unit and layer.** Whole-body thermoregulation is assigned to Layer 6, with core temperature Xi(t) as the selected state variable rather than the systemic layer as a whole. Behavioral heat seeking or avoidance belongs to Layer 7 and couples to this response.

**Viable set.** For illustration, let Vi(t) be prespecified as 36.5–37.5 °C for a defined measurement route, age group, time of day, activity state, and health context [42]. This is a functional example, not a universal normal range or the absolute limits of survival.

**Mismatch.** If d is the absolute distance to the nearest boundary and cold exposure lowers Xi(t) to 35.5 °C, Mi(t) = 1.0 °C. The success of the response is evaluated by whether this independently defined distance decreases.

**Corrective reduction.** Shivering, cutaneous vasoconstriction, and increased metabolic heat production return temperature toward the previously maintained viable range.

**Anticipatory and adaptive reduction.** Heat seeking before exposure illustrates response to projected mismatch. During infection, pyrogenic signaling can shift the defended range upward; movement toward that independently specified range is adaptive because it establishes a new thermal state. Fever therefore differs from uncontrolled hyperthermia [43].

**Failure and propagation.** Persistent hypothermia, hyperthermia, or excessive fever leaves unresolved mismatch and can impose metabolic and cardiovascular demands that propagate to organs, tissues, cells, behavior, and performance.

**Figure 3:**
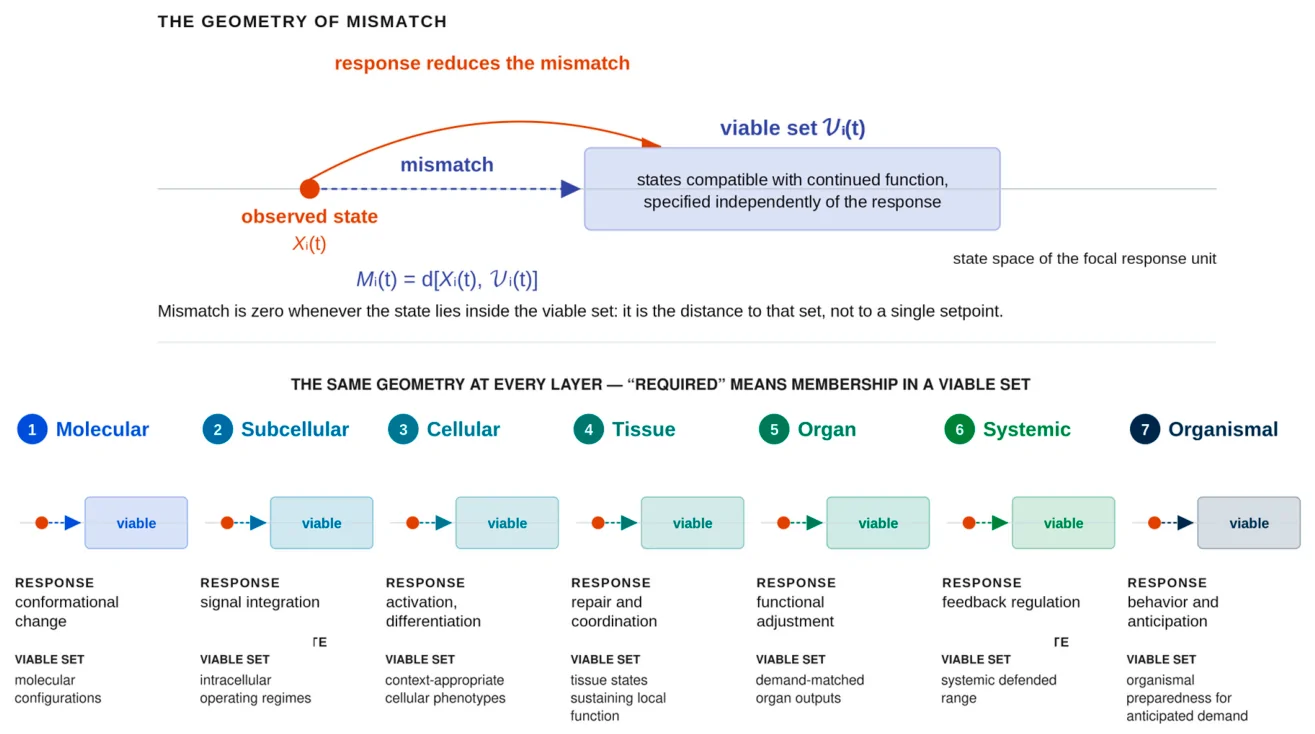
Mismatch reduction as the unifying principle of stratodynamics. At every biological layer, response can be understood as reduction of mismatch between the current state and an independently specified viable set compatible with continued function under prevailing conditions. ‘Required state’ is therefore shorthand for membership in that set, not a single target value. Layer-specific viable sets may comprise molecular configurations, intracellular operating regimes, context-appropriate cellular phenotypes, tissue states sustaining local function, demand-matched organ outputs, a systemic defended range, or organismal preparedness for anticipated demand. Equation (1), M_i_(t) = d[X_i_(t), V_i_(t)], defines mismatch for focal response unit i as the system-specific distance between its observed or projected state and its independently specified viable set. Mismatch is zero within the viable set and positive outside it; for example, body temperature outside its context-appropriate range has a mismatch corresponding to its distance from the nearest viable boundary [42,43]. The blue-to-green color progression distinguishes the seven biological layers, from molecular (blue) through subcellular, cellular, tissue, organ, and systemic levels (teal–green) to the organismal layer (dark blue). Orange denotes the observed state and the directional response toward the viable set, while the lightly shaded boxes represent the corresponding viable sets at each layer.

This shared mismatch-reduction logic is summarized in Figure 3.

## 5. The Stratification of Biological Response and Its Relation to Hierarchy Theory

Three questions define the theoretical foundation of Stratodynamics: what kind of stratification is proposed, why a layered partition is biologically plausible, and why seven layers are used. Hierarchy theory is examined as a comparator and source of terminology, not as a foundation from which stratodynamics is derived.

### 5.1. A Compositional Ordering of Response Scales, Not a General Hierarchy Theory

Any layered account of biology must state what its layers are layers of. Stratodynamics proposes a response-specific stratification: a compositional ordering of focal response units from molecular to organismal scale, not a universal anatomical taxonomy, a one-level-per-discipline scheme, or a general ontology of nature. A candidate stratum is distinguished by three criteria: recurring compositional scale, a characteristic response architecture, and sufficient empirical separability and reproducibility to be modeled and perturbed as a unit. The systemic and organismal strata are functional scales of integration rather than anatomical structures, confirming that assignment is response-specific rather than purely anatomical.

Salthe’s triadic representation of a focal level framed by lower-level initiating conditions and upper-level boundary conditions provides a useful comparison [44]. Stratodynamics does not equate its couplings with that triad. Upward, downward, and lateral coupling are defined as source–target dependencies that alter the probability, magnitude, timing, threshold, or trajectory of a response; they may be adjacent or nonadjacent and must be established empirically. Salthe’s model therefore helps clarify one possible form of cross-scale constraint, but it does not derive the seven strata or their response architectures.

Noble’s cardiac analysis demonstrates that cell-scale membrane potential and protein-scale ion-channel gating form a reciprocal dependency and that initial and boundary conditions are required to solve lower-scale equations [45]. Stratodynamics treats this as an instructive example of measurable cross-scale dependence, not as an axiom that no level of causation is privileged, nor as evidence that biological response is emergent. For each application, the direction, magnitude, delay, and mediator of influence remain empirical questions.

Stratodynamics therefore rejects the comprehensive “layer-cake” conception of levels—the uniform, one-level-per-discipline stratification associated with Oppenheim and Putnam [46]. Contemporary analyses emphasize that there is no widely accepted universal definition of biological levels and that local, heuristic, or purpose-relative accounts are often more defensible [47,48,49,50,51]. The strata used here are accordingly analytical categories for vertebrate response biology, not claims that nature is divided everywhere into the same discrete levels.

### 5.2. Why a Layered Partition Is Plausible: Partial Separability and Stable Intermediate Forms

Simon defined complex systems as many parts interacting in non-simple ways and described hierarchy and near-decomposability as common organizational features [52]. Stratodynamics does not adopt his broader proposition that the whole is more than the sum of its parts. It uses only the narrower, testable possibility that some response regimes show sufficiently stronger or more rapid internal coordination than cross-regime dependencies to be analyzed separately without being treated as independent. Because physiological timescales overlap, partial separability supports an analytical partition but does not determine the number or boundaries of the strata.

Simon’s stable intermediate form argument offers a plausibility rationale for why compositional organization can evolve and persist: disruption of one subassembly need not destroy the entire organism [52]. This does not prove modularity, derive the seven response strata, or make near-decomposability a universal property of vertebrate physiology. The seven-stratum scheme remains a provisional response-centered partition evaluated by the criteria stated below.

Natural selection is not assumed to act independently on each of the seven layers. It acts through heritable differences expressed in organismal survival and reproduction, while shaping lower-layer mechanisms and inter-layer couplings insofar as they contribute to the resulting phenotype. This framing also yields an evolutionary-medicine implication: a response that is protective during an acute challenge may impose a chronic cost when its duration, intensity, or predictive cue no longer matches the environment in which the response evolved. Historical constraint, trade-offs, and cue–environment mismatch may therefore generate persistent cross-layer incoherence. These are testable implications of the framework, not evidence that the seven layers constitute autonomous evolutionary units.

### 5.3. Criteria for a Layer, and Why Seven

The preceding analysis yields criteria for evaluating candidate stratum partitions rather than mechanically deriving a unique number. A scale qualifies as a distinct stratum of response when it satisfies three conditions: (i) compositional recurrence—it occupies a repeatedly encountered scale of focal response units in vertebrate organization; (ii) architectural distinctness—it exhibits a characteristic mode of state change not merely identical to that of adjacent scales; and (iii) empirical separability and reproducibility—its responses form a sufficiently regular regime to be measured, modeled, and perturbed as a unit [47,51]. Characteristic timing may support the third criterion, but adjacent strata need not have non-overlapping timescales or a monotonic fast-to-slow ordering.

Applied to the response biology of complex vertebrates, these criteria yield seven layers. The number is empirical and revisable, not fundamental. Reasonable alternatives can be evaluated against the same criteria: the subcellular layer might be divided into a signaling layer and an organellar layer, yielding eight; the tissue and organ layers might in some contexts be treated together, yielding six; and supraorganismal scales—populations, social groups, ecosystems—lie beyond the present, deliberately organism-scoped framework. Seven is proposed as the best current individuation for vertebrate physiology under the stated criteria, and the framework commits in advance to revising the count should evidence favor a different articulation. The scheme is thus provisional in its boundaries and count, but principled in the criteria that generate them.

The proposed partition spans the organism-scoped sequence from molecular response units to whole-organism response. It does not exclude candidate intermediate boundaries; those remain empirical under the stated criteria and Prediction 1.

### 5.4. Position Relative to Existing and Recent Multi-Scale Frameworks

Stratodynamics is not the first framework to treat biological organization across scales. Systems biology models interacting components and networks; multiscale physiology integrates selected spatial and temporal scales; network medicine analyzes disease networks and modules; and hierarchy theory examines levels, constraints, near-decomposability, and multilevel causation [44,45,51,52]. These frameworks are included as comparators because they overlap with aspects of stratodynamics, not because stratodynamics is derived from them. In particular, stratodynamics does not adopt non-additivity, emergence, robustness, modularity, network topology, or “no privileged level of causation” as its unifying principle. Its distinct proposal is to assign focal responses to provisional compositional strata, define mismatch relative to independently specified viable sets, formulate upward, downward, and lateral dependencies as perturbable relationships, and test whether persistent disease can involve failure to restore cross-stratum coherence. Homeostasis and allostasis remain established architectures of corrective and anticipatory regulation and can recruit mechanisms across several strata.

Among recent proposals, six are especially important comparators. First, Network Physiology and related adaptive-network approaches analyze time-varying coordination among physiological and organ systems and its reorganization across states [53,54]. Stratodynamics shares their emphasis on dynamic coupling but extends the analysis across seven prespecified molecular-to-organismal response strata, assigns characteristic response architectures to those strata, and defines mismatch relative to independently specified viable sets. Second, the multiscale competency architecture and related collective-intelligence account describe living systems as nested collectives that solve scale-appropriate problems and maintain goal-directed competencies across organizational levels [55,56,57]. This is closely aligned with upward and downward coordination in stratodynamics. The distinction is that stratodynamics specifies seven vertebrate response strata, assigns each a characteristic response architecture, and treats loss of coherence among those strata as a disease hypothesis.

Third, Kuang’s five-domain framework organizes whole-body function into operational physiological domains and emphasizes their integration [58]. Fourth, cellular viability-space theory formalizes survival and death as trajectories relative to viable regions of cellular state space [59]. These proposals sharpen two parts of stratodynamics: whole-body functions need explicit cross-domain coordination, and mismatch must be defined relative to independently specified viable states. Stratodynamics differs by organizing response compositionally from molecules to the whole organism and by applying viable-set mismatch across all seven strata rather than only at the cellular scale.

Fifth, the hallmarks of the pre-disease state frame disease onset as a critical transition marked by declining resilience and early-warning dynamics [60]. Sixth, the free-energy principle and active inference describe self-maintenance through prediction and action that limit variational free energy [34]. Pre-disease criticality supplies a useful dynamical language for impending loss of stability, whereas active inference is strongest for organism–environment regulation. Stratodynamics proposes a complementary internal architecture: it asks in which response stratum mismatch first appears, how it propagates upward, downward, or laterally, and whether coherence is restored before a critical transition becomes established disease.

Recent multiscale accounts of resilience and robustness seek to connect the temporal dynamics and plasticity of sub-organismal regulatory networks with organismal and higher-level outcomes [61]. This perspective is complementary to Stratodynamics: resilience and robustness characterize properties of response performance under disturbance, whereas stratodynamics specifies the layer-specific architectures and directional couplings through which persistence, recovery, or adaptive reorganization may be achieved or lost.

What stratodynamics adds is therefore not hierarchy, systems behavior, cross-scale interaction, or any other component in isolation. Its distinctive proposition is the joint organization of four elements: (i) scale-specific response architectures; (ii) mismatch reduction relative to prespecified viable sets; (iii) upward, downward, and lateral couplings that can be measured and perturbed; and (iv) a disease hypothesis in which persistence may result from failure to restore coherence among layers. Each element overlaps with established fields; the proposed advance lies in their prespecified integration and in the empirical commitments generated by that integration. The distinction from neighboring traditions can be stated precisely. Complex adaptive systems theory supplies emergence and self-organization but does not assign response units to a fixed compositional stratification; active inference supplies prediction and self-maintenance but is strongest at the organism–environment boundary and does not specify internal response architectures; multiscale computational physiology models cross-scale interaction quantitatively but does not claim one response logic recurring at every scale. Each therefore supplies one or two of the four elements above; none prespecifies all four jointly. A new term is justified only on that basis, and only insofar as the integration yields predictions its predecessors do not—which is the empirical burden of the framework. The comparison is summarized in Table 1.

Relative to quantitative multiscale and network models, stratodynamics presently lacks a unified parameterized implementation and prospective cross-layer validation. Relative to homeostasis and allostasis, its broader scope comes at the cost of reduced scale-specific quantitative precision. Its seven-stratum partition also remains provisional. These are limitations of the present framework, not limitations of the comparator theories.

## 6. The Seven Layers: Architecture and Exemplary Mechanisms

The previous section established that each layer of stratodynamics responds to its own form of mismatch and reduces that mismatch through its own architecture. The present section develops the seven layers in mechanistic detail. For each layer, the following are specified: the organizational scale at which the layer operates, the characteristic response architecture, the timescale over which response unfolds, exemplary mechanisms across biological systems, and the theoretical framework most precisely applicable to that layer.

The purpose of this section is to show that stratodynamics is not an abstract taxonomy of biological levels. Each layer corresponds to real biological structures, real mechanisms, and measurable processes operating on characteristic timescales. The seven layers are not separate compartments. They are functional strata of response. Each layer has its own architecture, but each is coupled to the others. Together they constitute the layered architecture through which living organisms detect, interpret, and respond to stimuli.

### 6.1. Layer 1: Molecular

**Scale**. Layer 1 operates at the scale of single molecules and small molecular complexes: receptors, enzymes, transcription factors, ion channels, structural proteins, RNA species, lipid mediators, and oligomeric assemblies. The unit of organization is the macromolecule or molecular complex in its native biochemical context.

**Architecture**. The architecture of Layer 1 is molecular-state transition. A molecule changes its configuration, occupancy, folding state, catalytic activity, localization, or interaction state in response to ligand binding, covalent modification, voltage, mechanical force, light, redox change, or another physicochemical perturbation. In this layer, the response architecture is compressed into the molecule itself. The molecule does not require a separate sensor, integrator, and effector in Cannon’s sense. Its binding surface, energy landscape, and conformational transition together constitute the response.

**Theoretical framework**. Layer 1 is most precisely described by molecular thermodynamics, statistical mechanics, chemical kinetics, structural biology, and allosteric theory. Conformational state distributions are governed by free-energy landscapes; ligand binding follows mass-action principles; transitions follow kinetic rules; and cooperative behavior is captured by allosteric models such as Monod–Wyman–Changeux and related modern extensions [62]. No homeostatic setpoint, allostatic prediction, or tissue program exists at this scale. Layer 1 is biological response at its thermodynamic and molecular ground.

In vertebrate medicine, Layer 1 is often where molecular specificity is easiest to measure but hardest to interpret alone. Hormone receptors, cytokine receptors, pattern-recognition receptors, ion channels, enzymes, transporters, and structural proteins all respond through changes in binding, conformation, activity, localization, or modification. Insulin binding to its receptor, calcium binding to calmodulin, oxygen binding to hemoglobin, glucocorticoid binding to its nuclear receptor, or microbial ligand binding to TLR4 are not systemic responses by themselves. They are molecular-state transitions that can become systemic only if they propagate through higher layers.

This distinction prevents an important interpretive error. A molecule may be necessary for a response without being sufficient to explain the response. A cytokine molecule can initiate signaling, but inflammation is not a molecule; it is a tissue program. Insulin can bind its receptor, but glucose regulation is not only receptor binding; it includes cellular uptake, liver output, pancreatic secretion, endocrine counter-regulation, feeding behavior, and organismal state. Layer 1 therefore provides causal entry points, but stratodynamics asks how those molecular events are translated upward and constrained downward.

### 6.2. Layer 2: Subcellular

**Scale**. Layer 2 operates at the scale of intracellular signaling networks, organelles, and regulatory modules within a single cell. The unit of organization is no longer a single molecule, but a coordinated intracellular system: kinase cascades, second-messenger systems, metabolic sensors, redox systems, organelle stress pathways, inflammasomes, proteostasis networks, mitochondrial networks, and transcriptional signaling modules.

**Architecture**. The architecture of Layer 2 is intracellular integration. Multiple Layer 1 events converge into signaling modules that amplify, filter, spatially organize, and temporally encode information. These modules may behave as thresholds, switches, oscillators, gradients, pulses, adaptive circuits, or spatially restricted signaling domains. Threshold crossing is a major feature of Layer 2, but not its only logic. Calcium signals, for example, often encode information through amplitude, duration, frequency, and localization; NF-κB signaling can encode information through nuclear translocation dynamics and oscillations; mitogen-activated protein kinase (MAPK) signaling may behave as graded or switch-like depending on cellular context.

**Exemplary mechanisms across species**. MAPK cascades, conserved from yeast to mammals, illustrate canonical Layer 2 architecture [63]. Receptor activation engages small GTPases and kinase cascades that amplify and transmit signals through MAP kinase kinase kinases, MAP kinase kinases, and MAP kinases. Depending on context, MAPK output may regulate proliferation, differentiation, stress response, survival, or apoptosis.

NF-κB signaling, central to inflammatory and immune responses across metazoans, illustrates Layer 2 integration of multiple upstream signals [64]. Toll-like receptor engagement, tumor necrosis factor α (TNF-α), IL-1β, reactive oxygen species, hypoxia, and cellular stress can converge on IκB kinase activation. IκB degradation permits NF-κB nuclear translocation and transcriptional activation. The response is not simply “on” or “off”; its amplitude, duration, oscillatory behavior, and crosstalk with other pathways shape cellular outcome.

Other canonical Layer 2 modules include mTOR nutrient sensing [65], the integrated stress response [66], and inflammasome assembly [67], which integrate metabolic, proteostatic, and inflammatory inputs, respectively.

**Theoretical framework**. Layer 2 is described by the systems biology of signaling networks, nonlinear dynamics, network motifs, control theory, ultrasensitivity, bistability, stochastic signaling, and spatial cell biology. Its recurring motifs include feedback loops, feed-forward loops, threshold modules, oscillators, pulse generators, adaptation circuits, and noise filters [68,69,70]. Layer 2 performs genuine intracellular computation, not in a cognitive sense, but in the biological sense of integrating multiple molecular inputs into coordinated cellular instructions.

In clinical and veterinary physiology, subcellular integration often explains why the same stimulus produces different outcomes in different conditions. A macrophage exposed to microbial products during energy deficit, hypoxia, glucocorticoid elevation, or tissue damage does not have the same signaling landscape as a macrophage in a resolving tissue. A mammary epithelial cell during early lactation does not have the same intracellular operating regime as the same cell during the dry period. The stimulus may be similar, but Layer 2 decides how that stimulus is interpreted inside the cell.

### 6.3. Layer 3: Cellular

**Scale**. Layer 3 operates at the scale of individual cells as integrated phenotypic units. The unit of organization is the cell itself, including its gene-regulatory networks, epigenetic state, metabolic program, cytoskeleton, membrane phenotype, secretory profile, motility, and capacity for division, death, or differentiation.

**Architecture**. The architecture of Layer 3 is cellular-state transition. A cell integrates intracellular signals, tissue context, developmental history, mechanical cues, metabolic state, and systemic signals, and responds by changing its phenotype or behavior. This may include activation, secretion, migration, proliferation, metabolic switching, polarization, apoptosis, senescence, or differentiation. In its most durable form, Layer 3 response is cell-fate commitment.

The gene-regulatory architecture of Layer 3 often involves multistable networks. Mutually reinforcing and mutually inhibiting transcriptional circuits create attractor states corresponding to distinct cellular phenotypes. A cell transitions from one state to another when integrated signals push it across a boundary in gene-expression state space. The Waddington landscape remains a useful conceptual image, but modern systems biology now describes such transitions through measurable changes in gene expression, chromatin accessibility, transcription-factor activity, and epigenetic memory.

**Exemplary mechanisms across species**. T-helper cell differentiation provides a clear example of Layer 3 architecture [71]. A naive CD4+ T cell receiving antigen and costimulation integrates the cytokine context of its environment. IL-12 favors Th1 differentiation through T-bet and IFN-γ; IL-4 favors Th2 differentiation through GATA3 and IL-4/IL-5/IL-13; TGF-β with IL-6 favors Th17 differentiation through RORγt and IL-17; TGF-β alone can favor regulatory T-cell development through FoxP3. These fates are not simple outputs of one signal. They are cellular-state transitions generated by integrated context.

Layer 3 is central to immune, inflammatory, regenerative, endocrine, and degenerative biology because cells are not passive containers of molecular pathways. They are integrated living units capable of changing phenotype. A neutrophil can become activated and migrate; hematopoietic cells can commit to different lineages [72]; a macrophage can adopt inflammatory, reparative, regulatory, or tissue-resident states [73]; a T cell can differentiate into effector or regulatory phenotypes; a fibroblast can become a myofibroblast [74]; and an epithelial cell can proliferate, migrate, secrete, or enter stress-associated states. These transitions are not merely biochemical changes. They are changes in cellular identity and function.

**Theoretical framework**. Layer 3 is most precisely described by multistable gene-regulatory networks, attractor dynamics, quantitative epigenetic landscapes, cell-state transition theory, and single-cell systems biology [75,76,77]. The key theoretical principle is that cells do not merely execute molecular signals; they integrate context and move among phenotypic states.

This cellular-state logic is highly relevant to chronic disease. Chronic inflammation may persist not only because inflammatory mediators remain elevated, but because cells become stabilized in states that continuously reinforce injury, fibrosis, vascular activation, or immune recruitment. In the mammary gland, epithelial, stromal, endothelial, and immune-cell states determine whether a response remains defensive, becomes damaging, or resolves. In the hoof, vascular, immune, fibroblast, keratinocyte, and pain-related cell states may determine whether a tissue returns to integrity or progresses toward chronic lesion formation. Layer 3 therefore links molecular triggers to durable biological memory.

### 6.4. Layer 4: Tissue

**Scale**. Layer 4 operates at the scale of multicellular tissues. The unit of organization is the tissue as a spatially organized collective of multiple cell types embedded in extracellular matrix, vascular networks, nerves, immune surveillance, mechanical forces, and bioelectric fields.

**Architecture**. The architecture of Layer 4 is the distributed multicellular program. Tissue responses are not reducible to the behavior of any single cell type. They require coordinated interactions among epithelial, mesenchymal, endothelial, immune, neural, stromal, and extracellular-matrix components. Communication occurs through soluble mediators, morphogen gradients, cytokines, growth factors, contact-dependent signaling, mechanical forces, matrix stiffness, oxygen gradients, metabolic gradients, and bioelectric coupling.

**Exemplary mechanisms across species**. Wound healing is a canonical Layer 4 process [78]. Hemostasis, inflammation, proliferation, and remodeling coordinate platelets, neutrophils, monocytes, macrophages, lymphocytes, fibroblasts, keratinocytes, endothelial cells, nerves, extracellular matrix, and local biochemical and bioelectric gradients. No single cell “heals” a wound. Healing is a tissue-level program.

**Inflammation is likewise a Layer 4 program**. Pathogen-associated and damage-associated signals initiate cytokine production, vascular activation, leukocyte recruitment, phagocyte activation, antimicrobial defense, and tissue remodeling. Resolution is not passive decay but active termination involving specialized pro-resolving mediators such as resolvins, protectins, maresins, and lipoxins [17,18]. These mediators limit neutrophil recruitment, promote efferocytosis, support macrophage phenotype transitions, enhance clearance, and permit restoration of tissue function. Failure of resolution is therefore a Layer 4 program-completion failure.

**Theoretical framework**. Layer 4 is described by developmental biology, tissue mechanics, reaction–diffusion theory, morphogen-gradient theory, bioelectric morphogenesis, wound-healing biology, inflammation and resolution biology, and regenerative biology [79,80,81]. No single theory yet integrates all these components. Stratodynamics locates them within one layer because they share the same scale: the tissue as a multicellular organized field.

Resolution biology is especially important at this layer. A tissue response must not only start; it must also stop in the correct way. Neutrophil recruitment, macrophage efferocytosis, lymphatic clearance, matrix remodeling, epithelial closure, vascular stabilization, and restoration of bioelectric and mechanical organization must be timed. When initiation occurs without resolution, repair becomes chronic inflammation, fibrosis, non-healing wounds, or recurrent tissue injury. In stratodynamic language, the tissue layer fails to complete mismatch reduction even if some molecular or cellular markers appear improved.

### 6.5. Layer 5: Organ

**Scale**. Layer 5 operates at the scale of organs as integrated functional units composed of multiple tissues. The unit of organization is the organ: a coherent anatomical and physiological structure delivering a defined function to the organism.

**Architecture**. The architecture of Layer 5 is integrated organ output. Organs contain multiple tissue compartments and functional units operating together. Nephrons, hepatic lobules, alveoli, pancreatic islets, lymphoid follicles, mammary alveoli, cardiac muscle units, and endocrine cell clusters are not isolated tissue programs. They are organized into organ-level outputs: filtration, secretion, metabolism, contraction, absorption, transduction, immune activation, reproduction, or nutrient delivery.

**Exemplary mechanisms across species**. The kidney is a canonical Layer 5 organ [82]. Nephrons operate in parallel to integrate glomerular filtration, tubular reabsorption, secretion, acid–base regulation, water handling, and electrolyte balance. The juxtaglomerular apparatus senses perfusion and sodium delivery, the macula densa participates in tubuloglomerular feedback, and endocrine inputs such as aldosterone, vasopressin, atrial natriuretic peptide, and angiotensin II calibrate renal output to systemic demand. The liver acute-phase response provides another organ-level output in which inflammation changes circulating proteins and systemic physiology [83].

The bone marrow illustrates Layer 5 hematopoietic output. During infection, inflammatory mediators and granulocyte colony-stimulating factor (G-CSF) drive emergency granulopoiesis, increasing neutrophil production and release into the circulation [84]. The response integrates stem-cell niches, stromal support, lineage commitment, cytokine gradients, vascular release, and systemic immune demand.

**Theoretical framework.** Layer 5 is described by classical organ physiology, integrative physiology, systems physiology, quantitative systems pharmacology, and organ-level control models [85]. Its theoretical language is functional integration: how tissues are coordinated into organ output and how that output is matched to systemic need.

The mammary gland illustrates why organ-level thinking cannot be replaced by either cellular or systemic thinking alone. Milk synthesis depends on epithelial cell metabolism, endocrine signals, nutrient supply, blood flow, immune surveillance, ductal integrity, stromal support, and animal-level energy balance. During mastitis, the same organ must simultaneously defend against microbes, protect tissue integrity, preserve or reduce secretion, manage pain, and communicate with systemic immunity. The disease cannot be understood fully as a bacterium, a cytokine, a somatic-cell count, or a milk-yield change alone. It is an organ-level disturbance embedded in layered response.

### 6.6. Layer 6: Systemic

**Scale**. Layer 6 operates at the scale of systemic regulated variables: temperature, arterial pressure, glucose, osmolarity, pH, oxygen tension, carbon dioxide tension, calcium, sodium, potassium, and other whole-body variables. The unit of organization is the regulated variable together with the sensors, integrators, and effectors that defend it.

**Architecture**. Layer 6 is the canonical site of Cannon’s homeostasis [4,5,6]. Its architecture consists of sensors that detect a regulated variable and effectors that are engaged as the variable crosses thresholds built into the sensing machinery, so that it settles within a defended range maintained by negative feedback. The variable is not fixed at a single value but held within a tolerated range. The nature of this ‘reference’ or setpoint, whether a stored value or a distributed consequence of effector thresholds, is taken up in future work.

**The setpoint and defended range are not always fixed**. They may be shifted by inter-layer signals arising from immune activity, circadian rhythms, reproductive state, stress, development, aging, pregnancy, lactation, or organismal anticipation. Fever illustrates this principle. During infection, pyrogenic cytokines such as IL-1β, IL-6, and TNF-α induce prostaglandin E_2_ signaling in the hypothalamic preoptic area, shifting the defended temperature range upward. The same Layer 6 thermoregulatory machinery then defends the new range through shivering, vasoconstriction, heat-seeking behavior, and metabolic heat production. When pyrogenic signaling falls, the defended range returns toward baseline, and the same architecture now promotes heat loss through vasodilation, sweating, and cooling behavior.

**Theoretical framework**. Layer 6 is described most precisely by Cannon’s homeostasis, cybernetics, feedback control, and classical systems physiology. This is the scale at which the homeostatic concept is strongest. Stratodynamics does not replace Cannon at Layer 6. It preserves Cannon’s framework and places it within a larger layered architecture.

Layer 6 remains the classical homeostatic layer and should be protected from conceptual dilution. Blood pH, osmolarity, temperature, oxygenation, carbon dioxide, calcium, sodium, potassium, glucose, blood pressure, and blood volume are not vague metaphors; they are measurable systemic variables with viable ranges. Vertebrate physiology depends on their defense. A cow, calf, dog, human patient, bird, or laboratory animal can tolerate many molecular or cellular variations, but cannot tolerate uncontrolled systemic collapse of oxygenation, pH, perfusion, temperature, or electrolyte balance. Major Layer 6 examples include central thermoregulatory control [86], pancreatic beta-cell electrical activity and insulin secretion in glucose regulation [87], and central osmosensation with systemic osmoregulation [88].

At the same time, Layer 6 is not independent. Fever shows that a defended systemic variable can be reset by tissue and immune signals. Lactation shows that calcium, glucose, lipids, amino acids, and water balance can be reorganized by reproductive and endocrine state. Exercise shows that blood pressure, ventilation, perfusion, and glucose use change before or during demand. Transition physiology in dairy cows shows that systemic regulation is challenged by simultaneous endocrine, inflammatory, metabolic, immune, and organ-output demands. Homeostasis is therefore preserved in stratodynamics, but it is placed inside a larger layered architecture.

### 6.7. Layer 7: Organismal

**Scale**. Layer 7 operates at the scale of the whole organism as an integrated, anticipatory, history-dependent unit. The unit of organization is the organism in relation to its internal state, external environment, prior experience, developmental history, circadian and seasonal cycles, immune memory, reproductive state, and behavioral possibilities.

**Architecture**. The defining architecture of Layer 7 is anticipatory pre-adjustment. The organism modifies lower layers in advance of expected demand. This requires a mapping between present cues and likely future conditions. In animals with complex nervous systems, the brain is the dominant integrator of this mapping. However, the principle is broader than the brain. Anticipatory regulation may also be encoded in circadian clocks, endocrine rhythms, immune memory, developmental programming, seasonal physiology, reproductive cycles, peripheral clocks, and evolutionary adaptation.

**Allostasis is the canonical Layer 7 framework**. Cortisol rises before waking in relation to circadian timing mechanisms, including suprachiasmatic nucleus rhythms [89]. Blood pressure rises before physical activity. Cephalic-phase insulin secretion begins before absorbed glucose enters the circulation. Immune responses are modified by prior infection, vaccination, trained immunity, and tissue-resident memory. Reproductive physiology anticipates breeding season in many vertebrate species through photoperiodic regulation [90]. Hibernating mammals pre-adjust metabolism, behavior, adipose stores, tissue function, and endocrine state before winter. Although outside the main vertebrate focus of this article, plant photoperiodic flowering illustrates why future domain-specific extensions of stratodynamics will require non-neural forms of anticipation [91].

**Theoretical framework**. Layer 7 is described by allostasis, allostatic load, predictive regulation, behavioral physiology, chronobiology, ecological physiology, and, in nervous systems, predictive-processing and free-energy frameworks [15,16,21,22,23]. Friston’s free energy principle may provide one mathematical description of neural organism-environment coupling, but it should not define the layer as a whole. The biological core of Layer 7 is anticipatory regulation: the capacity of the organism to prepare lower layers before the predicted demand arrives.

For veterinary sciences, this organismal layer is especially important because animals express disease not only through laboratory values but also through behavior, appetite, locomotion, social interaction, posture, pain avoidance, milk production, reproduction, and resilience. A lame cow, febrile calf, stressed sow, recovering horse, or chronically inflamed dog displays a whole-organism state. That state feeds downward into endocrine tone, immune readiness, organ output, tissue repair, cellular state, and molecular sensitivity. Layer 7 therefore gives stratodynamics its strongest bridge from physiology to animal welfare and clinical observation.

## 7. Inter-Layer Coupling: How Seven Layers Become One Response

The previous section described the seven layers of stratodynamics as architecturally distinct. Each layer has its own scale, components, timescale, and mode of mismatch reduction. This distinction is necessary, but it creates an immediate question: if the layers are different, how do they produce one coherent biological response?

The answer lies in coupling: demonstrable dependencies among response units at lower, higher, or the same stratum.

This section develops the architecture of inter-layer coupling. Its central claim is that biological response is generated at the interfaces among layers as much as within the layers themselves. The couplings are where local events become systemic, where systemic states reshape local events, where timing across scales is coordinated, and where many chronic diseases may become self-sustaining.

Because coupling is central to the framework, it is defined here mechanistically rather than used as a general synonym for interaction. **Coupling** denotes a demonstrable dependency through which the state or response of one unit or layer alters the probability, magnitude, timing, threshold, or available trajectories of another. The influence may be mediated by biochemical signals, metabolites, energy, matter, electrical activity, mechanical forces, or information-bearing patterns, or it may be imposed through constraints and boundary conditions. Coupling is thus not a single mechanism, but a class of dependencies unified by their effect: the response of one unit becomes a determinant of the response of another. Evidence of coupling should derive from a controlled perturbation or, when intervention is unavailable, from temporally ordered and appropriately conditioned measures of directed dependence. Temporal succession or contemporaneous correlation alone is insufficient, because either may arise from a shared upstream driver.

These directional classifications do not claim that cross-scale interaction is novel; signal transduction, systems integration, and hierarchical control already describe such phenomena. Stratodynamics instead integrates directed dependencies with mismatch propagation, layer-specific response architectures, thresholds, and disease-related loss of coherence.

**Figure 4:**
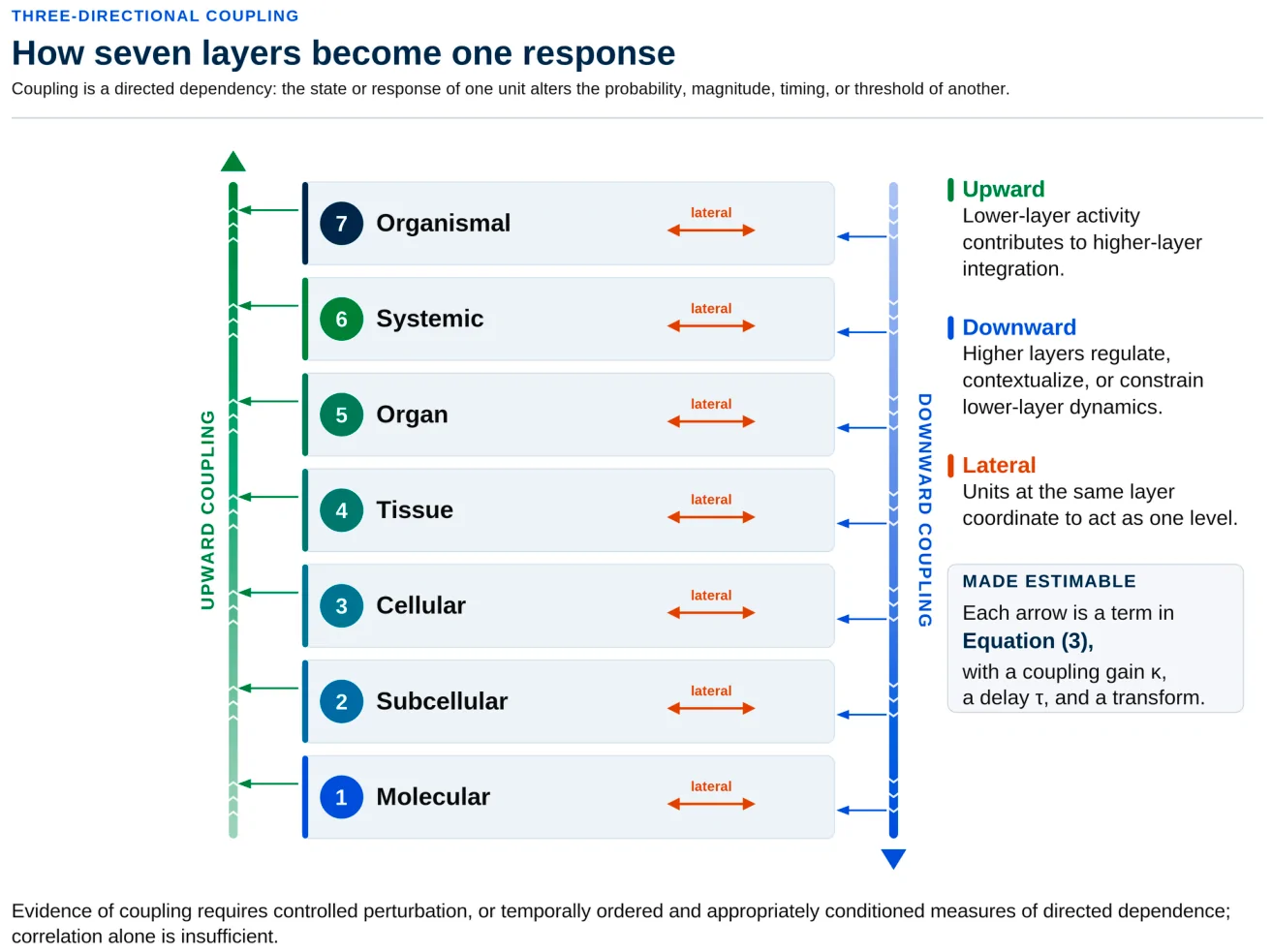
Three-directional coupling among stratodynamic layers. Upward coupling allows molecular and cellular events to propagate toward tissue, organ, systemic, and organismal responses. Downward coupling allows organismal state, systemic context, and prediction to shape organs, tissues, cells, signaling pathways, and molecular readiness. Lateral coupling coordinates components within each layer. Reading the figure: the seven strata are stacked with Layer 1 at the base. The ascending channel on the left denotes upward coupling and the descending channel on the right denotes downward coupling, with arrowheads marking direction of influence; the paired horizontal arrows within each band denote lateral coupling among units at the same scale. Every arrow corresponds to a term in Equation (3), with a signed coupling gain, a delay, and a transform from source to target, so that each depicted dependency is in principle estimable and perturbable rather than merely illustrative. Layer colors match Figure 2 and distinguish the seven strata; green denotes upward coupling, blue denotes downward coupling, and red denotes lateral coupling.

The three-directional coupling architecture is summarized in Figure 4.

The response of a unit therefore depends not on its own mismatch alone but on its coupling to other units—at lower, higher, and the same layer—and on the system’s history, which may be written

(2) $$R_{i}(t)=F_{i}[M_{i}(t),\;C_{i}(t;\;Z_{-i}(t)),\;H_{i}(t)]$$

where *F_i_* is the unit-specific response function, **Z***_−i_*(t) denotes the relevant collection of state and response variables from coupled source units within and across layers, excluding the focal response being modeled, *C_i_*(t;) is the potentially time-dependent coupling functional that maps these influences onto the response of unit *i*, and *H_i_*(t) represents history dependence. Where required, **Z***_−i_*(t) may include lagged state or response trajectories. The functional *C_i_* encompasses upward, downward, and lateral coupling and is not assumed to be linear, additive, or time-invariant. A transition may occur when the combined dynamics cross a layer-specific or system-wide threshold; in other systems the transition may be gradual or probabilistic.

For a single prespecified source–target relationship, Equation (2) can be given a minimal dynamical specification through the following local approximation:

*dX_i_*(*t*)/*dt* = *f_i_*[*X_i_*(*t*), *M_i_*(*t*), *H_i_*(*t*)] + *κ_ji_ g_ji_*[*X_j_*(*t* − *τ_ji_*)] (3)

Here, κ_ji_ is the signed coupling gain from source unit j to target unit i, τ_ji_ is the coupling delay, and g_ji_ transforms the source signal into the input received by the target. Attenuation, exaggeration, reversal, or mistiming can therefore be represented by changes in the magnitude or sign of κ_ji_ or in τ_ji_ relative to an independently specified reference. Equation (3) is a local, system-specific approximation rather than a universal kinetic law. Its purpose is to convert a verbal coupling claim into estimable parameters that can be perturbed and compared with a null or alternative model. This formulation is compatible with adaptive-network approaches, in which both node dynamics and coupling relations may change with internal state or external perturbation [54]. A biological interpretation of the general and local coupling equations is provided in Box 3.

Box 3 **Biological interpretation of the general and local coupling equations.**

Equations (2) and (3) are model templates rather than parameterized biological laws. The following wound-healing examples show how their symbols can be assigned to measurable biological quantities without implying fixed numerical values.

**Equation (2): integrated response of a focal biological unit.**

*R_i_*(*t*) = *F_i_*[*M_i_*(*t*), *C_i_*(*t*; *Z*_−_*_i_*(*t*)), *H_i_*(*t*)]

Equation (2) states that the response of a focal biological unit may depend on more than its own mismatch. It may also depend on signals, conditions, or constraints arising from other response units and on its previous biological history. Consider epithelial cells at the margin of a cutaneous wound as focal response unit i. Their response R_i_(t) may include migration, proliferation, and restoration of the epithelial barrier. The mismatch M_i_(t) is the remaining difference between the present epithelial state and the state required for barrier restoration.

The coupled variables Z_−i_(t) may include macrophage activation, fibroblast behavior, endothelial perfusion, extracellular-matrix organization, neural signals, oxygen availability, circulating hormones, and systemic metabolic state. The coupling functional C_i_ represents how these upward, downward, and lateral influences are integrated into the epithelial response. The history term H_i_(t) may include previous injury, prolonged inflammation, chronic hyperglycemia, prior infection, aging, or epigenetic changes that alter present repair capacity. Thus, two wounds with a similar epithelial defect may show different repair responses because their surrounding cellular activity, tissue environment, systemic condition, and biological history differ.

**Equation (3): one prespecified source–target coupling.**

*dX_i_*(*t*)/*dt* = *f_i_*[*X_i_*(*t*), *M_i_*(*t*), *H_i_*(*t*)] + *κ_ji_ g_ji_*[*X_j_*(*t* − *τ_ji_*)]

Equation (3) isolates one specific coupling from source unit j to target unit i. In the wound-healing example, source unit j may be the macrophage pro-resolution response and target unit i may be epithelial wound closure. The first term describes the epithelial response arising from its present state, remaining mismatch, and history. The second term describes how an earlier macrophage response changes the subsequent rate or trajectory of epithelial repair.

When Xi is defined as the extent of epithelial closure and g_ji_ is nonnegative, the coupling gain κ_ji_ represents the direction and strength of the source–target influence: a positive gain promotes repair, a value near zero indicates little measurable influence, and a negative gain inhibits repair. The delay τ_ji_ represents the time required for the macrophage response to affect the epithelium. The transformation function g_ji_ allows the source signal to act proportionally, through a threshold, by saturation, or through another system-specific relationship.

Coupling dysfunction may therefore appear as attenuation, when the source produces too little target response; exaggeration, when the response is excessive; mistiming, when the influence arrives too early or too late; reversal, when a normally supportive influence becomes inhibitory; or ectopic activation, when the response occurs in an inappropriate location or physiological context. In each application, the biological units, measured variables, units of measurement, functional forms, coupling gains, delays, viable references, and decision criteria must be defined in advance and estimated from experimental or longitudinal data.

Coupling dysfunction is proposed as one mechanism contributing to chronic disease and is defined operationally rather than by a universal numerical cutoff, because the relevant variables and ranges differ among biological systems. Within a defined system, coupling dysfunction may be identified, relative to an independently specified viable reference dependency, baseline, or null model, as attenuation, exaggeration, mistiming, reversal, or ectopic activation of the expected dependency between response units or layers. Although universal numerical cutoffs would be inappropriate, the relevant reference dependency and decision criterion must be specified in advance for each application.

### 7.1. Upward Coupling: From Molecular Event to Organismal Response

Bacterial infection illustrates upward coupling clearly. Lipopolysaccharide binding to the Toll-like receptor 4 (TLR4)–MD-2 receptor complex is a molecular event. That event activates intracellular signaling through MyD88- and TRIF-dependent pathways. These pathways induce cellular inflammatory programs in macrophages, dendritic cells, epithelial cells, and endothelial cells. Those cellular responses coordinate tissue inflammation, vascular activation, leukocyte recruitment, and antimicrobial defense. Tissue-derived cytokines alter organ function, including hepatic acute-phase synthesis and bone marrow granulopoiesis. Organ outputs change systemic variables, producing fever, altered glucose metabolism, leukocytosis, and endocrine responses. Finally, systemic signals alter organismal behavior, producing sickness behavior, rest, anorexia, pain sensitivity, and immune memory [92]. A microbial molecule has become a whole-organism response.

### 7.2. Downward Coupling: From Organismal State to Molecular Readiness

The molecular state of a cell is always embedded in a larger biological context. Circadian phase changes gene expression and receptor sensitivity in peripheral tissues. Stress history alters glucocorticoid tone, autonomic output, immune readiness, and inflammatory thresholds. Pregnancy and lactation reshape metabolism, endocrine sensitivity, vascular supply, mammary epithelial activity, bone-mineral dynamics, and immune responses. Infection shifts organ function and systemic variables, which then alter the behavior of cells throughout the body.

In veterinary physiology, downward coupling is visible whenever the same local insult produces different outcomes in different animals or at different physiological stages. The same microbial challenge to the mammary gland may be mild in a resilient cow, severe in a metabolically stressed cow, or recurrent in a cow whose tissue resolution is impaired. The same mechanical load on the hoof may be tolerated under one systemic state and become damaging under another. The same inflammatory mediator may produce different cellular responses in early lactation, late lactation, pregnancy, growth, aging, or chronic stress. The lower layers do not simply receive stimuli; they receive stimuli within a downwardly shaped organismal condition.

Downward coupling also explains why treatment directed at one local target may fail when the systemic or organismal context remains unfavorable. Blocking one mediator, killing one pathogen, or correcting one blood variable may be necessary, but durable recovery may require restoring the higher-level conditions that allow tissues to resolve, organs to normalize output, and cells to leave pathological states. This is not an argument against targeted therapy. It is an argument for locating targeted therapy within the layered context that determines whether the target can return to coherence.

### 7.3. Lateral Coupling: Coordination Within a Layer

Examples of lateral coupling include cooperative molecular reactions; crosstalk among intracellular pathways; communication among immune, epithelial, stromal, endothelial, and neural cells; coordination of vascular, matrix, inflammatory, mechanical, and bioelectric tissue processes; integration of organ compartments; joint regulation of systemic variables; and coordination of behavior, appetite, sleep, reproduction, and environmental anticipation.

### 7.4. Spatial and Temporal Coupling

Together, upward, downward, and lateral coupling gives biological response a three-dimensional architecture. The first dimension is vertical, from molecular to organismal scales; the second is horizontal, through coordination within each layer; and the third is temporal, through the different rates at which layers respond, adapt, resolve, or remember. A complete description must therefore identify which layers are engaged, how components within each layer are coordinated, and how the timing and delay of events unfold across layers. A receptor, cytokine, cell type, organ, or systemic variable may be essential, but represents only one point in this architecture. Timing is part of coherence rather than a secondary detail. Neutrophil recruitment is useful early in infection but damaging if prolonged; fibroblast activation supports repair but becomes pathological when myofibroblasts persist; fever can aid host defense but becomes harmful when excessive; and reduced appetite can conserve energy acutely while worsening prolonged negative energy balance. Many chronic disorders may therefore begin with an appropriate response that fails to transition: inflammation does not resolve, repair does not remodel, cellular activation does not return to quiescence, organ adaptation becomes maladaptation, or systemic compensation becomes burden.

### 7.5. Coupling Failure and Disease

The third central claim of stratodynamics is that many diseases, especially chronic, multifactorial, relapsing, and poorly resolving diseases, preferentially involve failures of inter-layer coupling. This claim should be understood as a testable hypothesis, not as an established universal law.

Single-layer origins clearly exist. Monogenic enzyme defects, channelopathies, mitochondrial DNA mutations, protein misfolding diseases, toxin-induced injury, trauma, nutritional deficiency, and certain infections may begin primarily within one layer. Once initiated, however, even these disorders propagate across layers. A molecular enzyme defect can become cellular stress, tissue injury, organ dysfunction, systemic disease, and organismal disability. Stratodynamics therefore does not deny single-layer disease origins; it asks how such origins propagate and how they become stabilized across layers.

The more distinctive claim is that chronic multifactorial diseases often persist because couplings fail to restore coherence. Each layer may operate reproducibly within its ordinary physiological range, but coupling requires translation across scales. Molecular signals must become cellular decisions. Cellular decisions must become tissue programs. Tissue programs must become organ outputs. Organ outputs must be integrated into systemic variables. Systemic variables must update organismal state. Each translation can fail by being too weak, too strong, too slow, too prolonged, mistimed, mislocalized, or improperly resolved.

Chronic inflammation can be framed as failure of tissue-level resolution coupling: inflammatory initiation occurs, but the transition to resolution, repair, and restoration is incomplete [17,18]. Type 2 diabetes can be framed as persistent mismatch among organismal prediction, systemic glucose regulation, pancreatic islet output, hepatic glucose production, adipose tissue signaling, skeletal muscle uptake, cellular nutrient sensing, and molecular insulin signaling. Autoimmunity can be framed as a failure that begins in cellular selection, tolerance, or immune memory, but becomes disease through coupling to tissue injury, organ dysfunction, systemic inflammation, and organismal state. Sepsis can be framed as catastrophic dyscoupling between tissue-level inflammatory amplification and systemic regulation. Chronic non-healing wounds can be framed as failure of the tissue program to transition properly through inflammation, proliferation, remodeling, vascularization, innervation, matrix organization, and bioelectric restoration.

These examples are not offered as proven localizations. They are hypotheses generated by the framework. Their value lies in making disease origins and disease persistence experimentally tractable. A layer-resolved approach would ask: where did the mismatch originate, how did it propagate, which coupling failed to resolve it, and which layer now maintains the pathological state?

If this view is borne out, the clinical implication is significant. Diagnosis would not ask only which molecule, cell type, organ, or systemic variable is abnormal. It would also ask which coupling among layers has failed. Therapy would not aim only to suppress the most visible downstream marker. It would aim to restore coherence across the relevant layers. In chronic disease, durable recovery may require correcting timing, scaling, communication, and resolution across layers, not merely blocking one mediator.

This clinical logic remains a research program. It requires layer-resolved diagnostics, longitudinal sampling, multi-omics integrated with physiology, tissue-level imaging, organ function measures, systemic variables, behavioral data, and computational models capable of mapping causality across scales. Stratodynamics provides the conceptual architecture for that program; the empirical work remains to be done.

**Figure 5:**
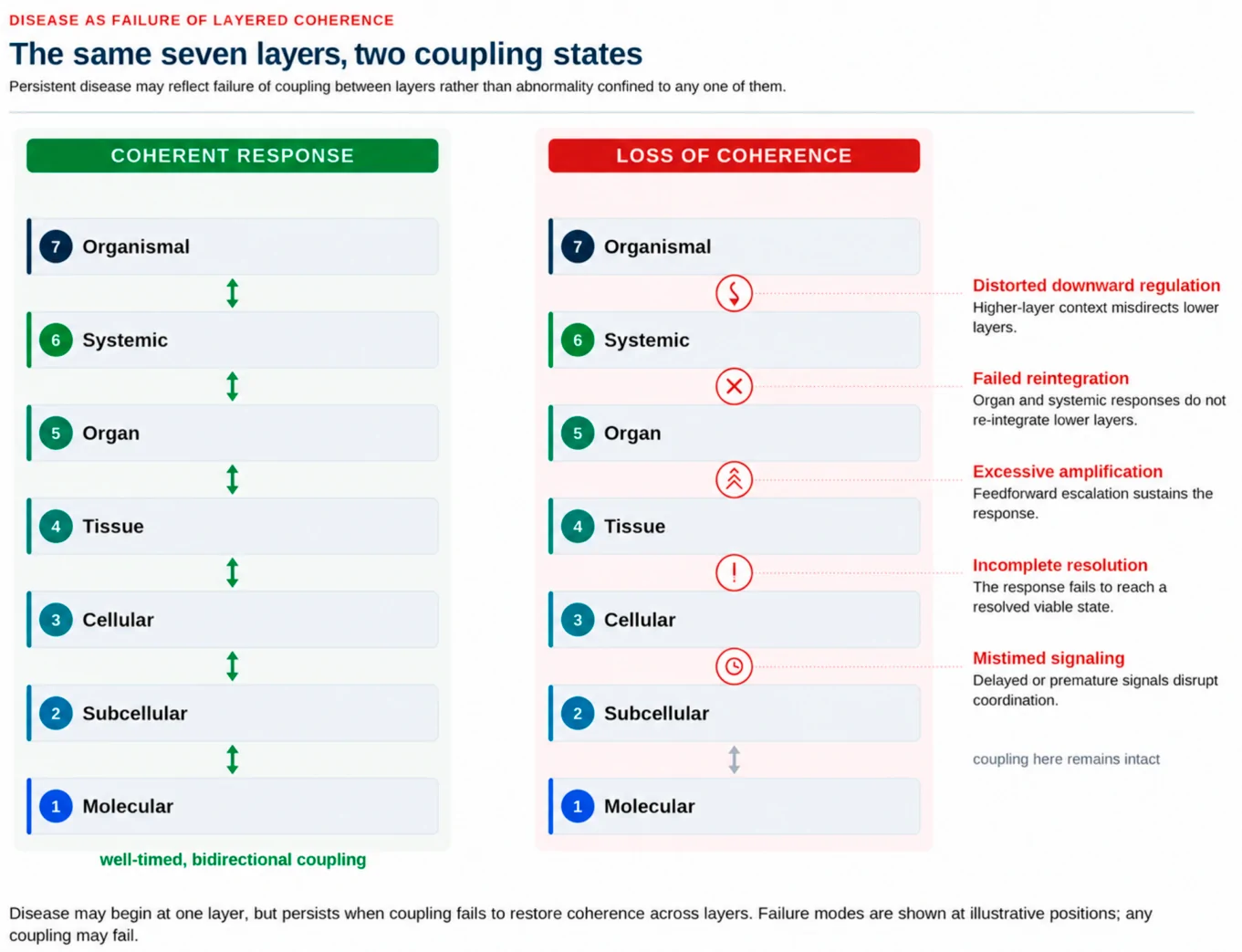
Disease as failure of layered coherence. Stratodynamics proposes that many chronic, multifactorial, relapsing, and poorly resolving diseases persist not only because one molecule, cell, organ, or systemic variable is abnormal, but because coupling among layers fails to restore coherence. Pathology may be sustained by mistimed signaling, incomplete resolution, excessive amplification, distorted downward regulation, or failure of organ and systemic responses to re-integrate tissue and cellular states. Reading the figure: the same seven strata are shown twice. On the left, intact bidirectional arrows between adjacent strata denote well-timed coupling. On the right, the annotated symbols mark five modes by which a coupling may fail, each placed at an illustrative rather than a fixed position; the grey arrow indicates that coupling elsewhere in the same system may remain intact, so that failure is selective rather than global. Disease may originate within one stratum, but on this account, it persists when coupling fails to restore coherence.

Major modes of failed layered coherence are summarized in Figure 5.

### 7.6. Layer Boundaries Are Real but Permeable

A final implication of the coupling architecture is that layer boundaries are real but permeable. Each layer has a characteristic scale, architecture, and timescale, but biological entities often participate in more than one layer depending on the question being asked.

The same molecule can therefore carry different meanings at different layers. The layer is not defined by the object alone, but by the role the object plays in an organized response. This is why stratodynamics is not a simple anatomical hierarchy. It is a response-specific stratification across compositional scales.

### 7.7. Worked Example: Wound Healing Across All Seven Layers

A cutaneous wound illustrates the full stratodynamic architecture in compressed form. Molecular clotting, fibrin formation, receptor binding, cytokines, and growth factors initiate the response. Intracellular signaling pathways translate these cues into platelet activation, leukocyte recruitment, macrophage transition, fibroblast activation, Notch-coordinated angiogenesis [93], and keratinocyte migration. At the tissue layer, hemostasis, inflammation, proliferation, remodeling, bioelectric guidance, matrix organization, and resolution are coordinated. At the organ layer, the skin progressively restores barrier function, microbial defense, sensation, thermoregulation, and mechanical protection. Systemic metabolism, endocrine adjustment, immune mobilization, and organismal behavior then support and protect the repair process.

**Figure 6:**
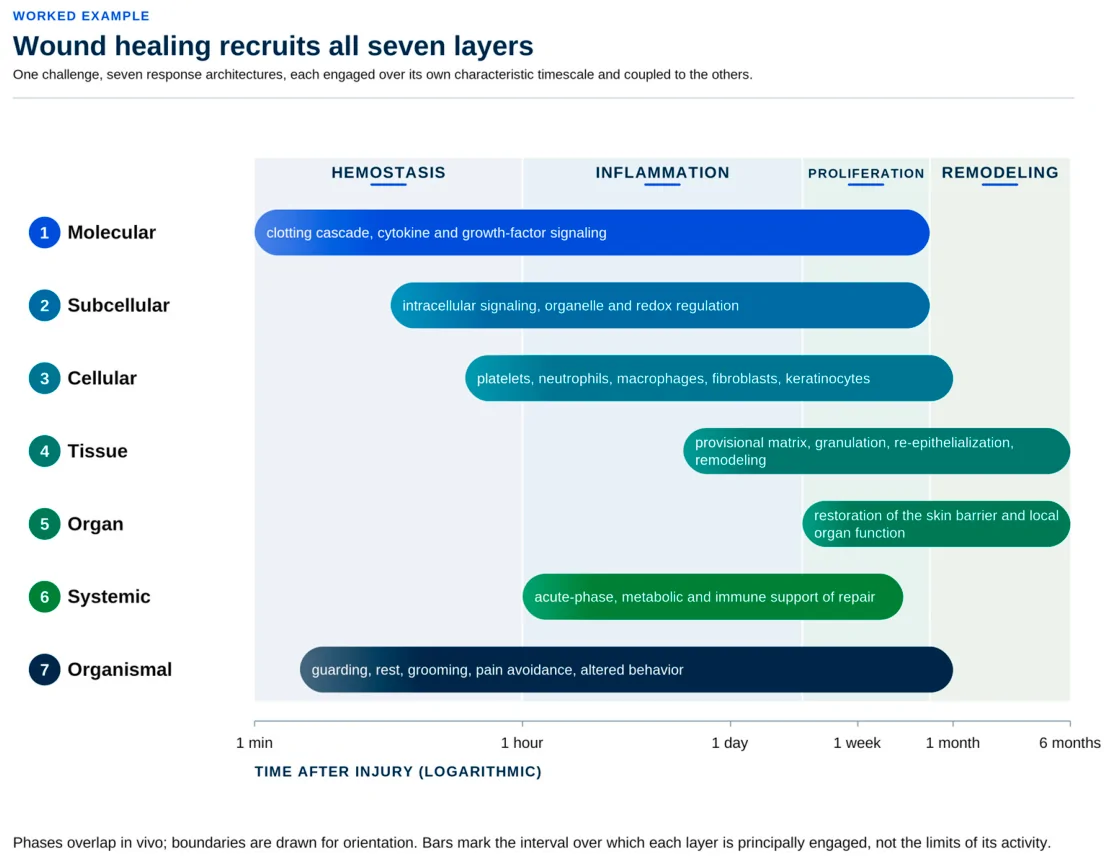
Wound healing as a stratodynamic response. A cutaneous wound recruits all seven layers of biological response. Molecular clotting and mediator signals activate intracellular pathways; cells migrate, proliferate, and change phenotype; tissues coordinate inflammation, repair, bioelectric guidance, and remodeling; the skin restores barrier function; systemic metabolism and immunity support repair; and organismal behavior protects the damaged site. Reading the figure: strata are shown as horizontal lanes and time after injury runs on a logarithmic axis from minutes to months, with the classical phases of repair indicated as background bands. Each bar marks the interval over which its stratum is principally engaged, not the limits of its activity; phases overlap in vivo and the boundaries are drawn for orientation. The staggered but broadly overlapping bars illustrate that strata operate on different characteristic timescales while remaining concurrently active and coupled.

The wound-healing example is summarized in Figure 6.

A cutaneous wound recruits the full stratodynamic architecture in a single coordinated response.

No single layer explains wound healing. Molecular events are necessary but insufficient. Cellular activation is necessary but insufficient. Tissue programs are necessary but insufficient. Organ function, systemic support, and organismal behavior are all required. Healing is the coupled activity of all seven layers.

## 8. Stratodynamics in Veterinary and Medical Sciences

The primary value of stratodynamics is not that it adds another term to physiology, but that it gives veterinary and medical sciences a practical way to organize complex biological responses. Clinicians and biomedical researchers often encounter conditions in which one marker, one cell type, one organ, or one pathway cannot explain the whole disease. The animal or patient presents as an integrated living system. Stratodynamics provides a language for asking how molecular events, cellular states, tissue programs, organ outputs, systemic variables, and organismal behavior are connected in that case.

This is particularly important for diseases of resilience. Some animals experience major physiological challenge and recover. Others experience similar challenge but develop chronic inflammation, metabolic instability, organ dysfunction, poor production, impaired fertility, recurrent infection, pain, or poor welfare. The difference may not lie in the presence or absence of one stimulus, but in whether the layers remain coordinated under challenge. In this sense, stratodynamics is a theory not only of disease, but also of resilience.

### 8.1. Worked Example: Transition Physiology in the Dairy Cow

The transition dairy cow provides a second worked example because the onset of lactation is a predictable, high-demand state transition rather than a simple return to the dry-period condition. The focal challenge includes calving, initiation of milk synthesis, uterine recovery, altered microbial exposure, and a rapid increase in nutrient and calcium demand. Successful adaptation therefore represents predominantly adaptive mismatch reduction: coordinated movement toward a new viable lactational state. The seven layers can be specified as follows.

**Layer 1—Molecular.** Ligand–receptor binding, enzyme-state changes, ion binding, and mediator interactions alter the activity of insulin, growth-hormone, catecholamine, glucocorticoid, parathyroid-hormone, cytokine, and innate-immune pathways. The focal mismatch concerns whether molecular states and sensitivities are appropriate to the emerging lactational demand.

**Layer 2—Subcellular.** Hepatocytes, mammary epithelial cells, adipocytes, myocytes, and immune cells integrate nutrient, calcium, redox, inflammatory, endoplasmic-reticulum, and mitochondrial signals through networks that include AMPK, mTOR, NF-κB, calcium handling, and organelle stress responses. The response problem is intracellular coordination rather than the concentration of any single mediator.

**Layer 3—Cellular.** Mammary epithelial cells enter a secretory state; hepatocytes increase gluconeogenic and lipid-handling activity; adipocytes mobilize stored energy; neutrophils, monocytes, macrophages, lymphocytes, and endometrial cells alter activation, trafficking, and resolution states. Cellular adaptation fails when these state transitions are insufficient, excessive, mistimed, or energetically unsustainable.

**Layer 4—Tissue.** Mammary tissue expands secretory function and barrier defense, the uterus undergoes involution and repair, placental tissues must detach and resolve inflammation, adipose tissue remodels during lipid mobilization, and the rumen epithelium adapts to the postpartum diet. Tissue-level mismatch is reduced when inflammatory, vascular, structural, and reparative programs proceed with appropriate localization and termination.

**Layer 5—Organ.** The mammary gland establishes milk output; the liver coordinates gluconeogenesis, ketogenesis, lipid oxidation, and lipoprotein export; the uterus restores structural and reproductive function; and the gastrointestinal tract, kidney, bone, and endocrine organs contribute to nutrient and mineral supply. Each organ must match its integrated output to the demands imposed by the transition.

**Layer 6—Systemic.** Circulating ionized calcium, glucose, non-esterified fatty acids, ketone bodies, acid–base balance, endocrine signals, acute-phase proteins, temperature, and immune mediators must remain within context-appropriate ranges. These regulated variables are coupled rather than independent, so correction of one variable may be insufficient if inflammatory, metabolic, and mineral regulation remain discoordinated.

**Layer 7—Organismal**. Feed intake, rumination, activity, rest, social behavior, pain avoidance, stress responsiveness, and maternal behavior integrate the cow’s physiological history and anticipated demand. Prepartum endocrine and behavioral adjustments can prepare lower layers for calving and lactation, whereas reduced intake, sustained stress, pain, or disrupted behavior can constrain recovery downward across the strata.

This worked example generates a testable layered model rather than a retrospective relabeling of familiar disorders. Before sampling, investigators would specify the response unit and viable set at each included layer, the expected direction and timing of selected couplings, and the clinical or functional endpoint. Hypocalcemia, ketosis, metritis, mastitis, retained placenta, displaced abomasum, and lameness remain clinically useful diagnoses; the stratodynamic hypothesis is that susceptibility, clustering, or persistence of these outcomes may be better predicted when mistimed or weakened cross-layer dependencies are included than when the same measurements are analyzed only as independent abnormalities.

### 8.2. Mastitis, Lameness, and Chronic Tissue Disease

Mastitis can be interpreted as a stratodynamic event because the visible disease is a mammary-gland disorder, but the response includes all seven layers. Microbial ligands and host mediators act at the molecular layer; epithelial and immune cells integrate danger signals intracellularly; leukocytes, epithelial cells, endothelial cells, fibroblasts, and stromal cells change state; the mammary tissue coordinates inflammation, barrier defense, edema, pain, and resolution; the gland alters milk secretion and composition; systemic variables and acute-phase responses are engaged; and the cow changes behavior, appetite, and production. Persistent or recurrent mastitis may therefore reflect not only pathogen exposure, but failure of coupling among tissue resolution, organ function, systemic metabolism, and organismal resilience.

Lameness and hoof lesions can be read in the same way. A hoof lesion is often the last visible event in a longer chain. Metabolic stress, inflammatory tone, vascular function, tissue perfusion, epithelial integrity, mechanical loading, pain behavior, locomotion, immune readiness, and repair capacity may all contribute. Stratodynamics does not deny local anatomy or mechanics. Rather, it places the hoof within the whole animal. The question becomes whether the hoof tissue remained coherent with systemic inflammatory-metabolic state, vascular supply, organ-level nutrient allocation, and organismal movement behavior.

### 8.3. Medical Translation: Chronic Inflammation, Metabolism, and Repair

The same reasoning can be extended to human and comparative medicine. Chronic inflammatory disease may persist because tissue-level resolution does not properly communicate with cellular phenotype, vascular remodeling, organ function, systemic inflammatory tone, and organismal stress state. Metabolic syndrome may reflect persistent mismatch among organismal prediction, feeding behavior, systemic glucose regulation, liver output, adipose tissue signaling, skeletal muscle uptake, cellular nutrient sensing, and molecular insulin signaling. Non-healing wounds may reflect failure of temporal coupling among inflammation, vascularization, epithelial migration, matrix remodeling, innervation, microbial control, and systemic metabolic support. These readings are hypotheses generated by the framework, offered as candidate explanations to be tested; they do not displace conclusions established from data. Each is consistent with published observations—active resolution programs and their failure in chronic inflammation [17,18,30], persistence of disease mechanisms during apparent remission [40], and the phased, timing-dependent character of repair [78]—but consistency is not demonstration. None of these interpretations has yet been tested prospectively as a stratodynamic prediction, and each is stated so that it could be refuted by the falsifiable designs specified later.

This approach does not replace molecular diagnosis, imaging, pathology, or clinical biomarkers. It organizes them. A biomarker becomes more informative when its layer is known. A cytokine measured in blood may indicate systemic inflammation, but the clinically relevant failure may lie in tissue resolution. Stratodynamics therefore encourages multi-layer diagnosis rather than single-marker interpretation.

### 8.4. Translational Questions Generated by Stratodynamics

**Figure 7:**
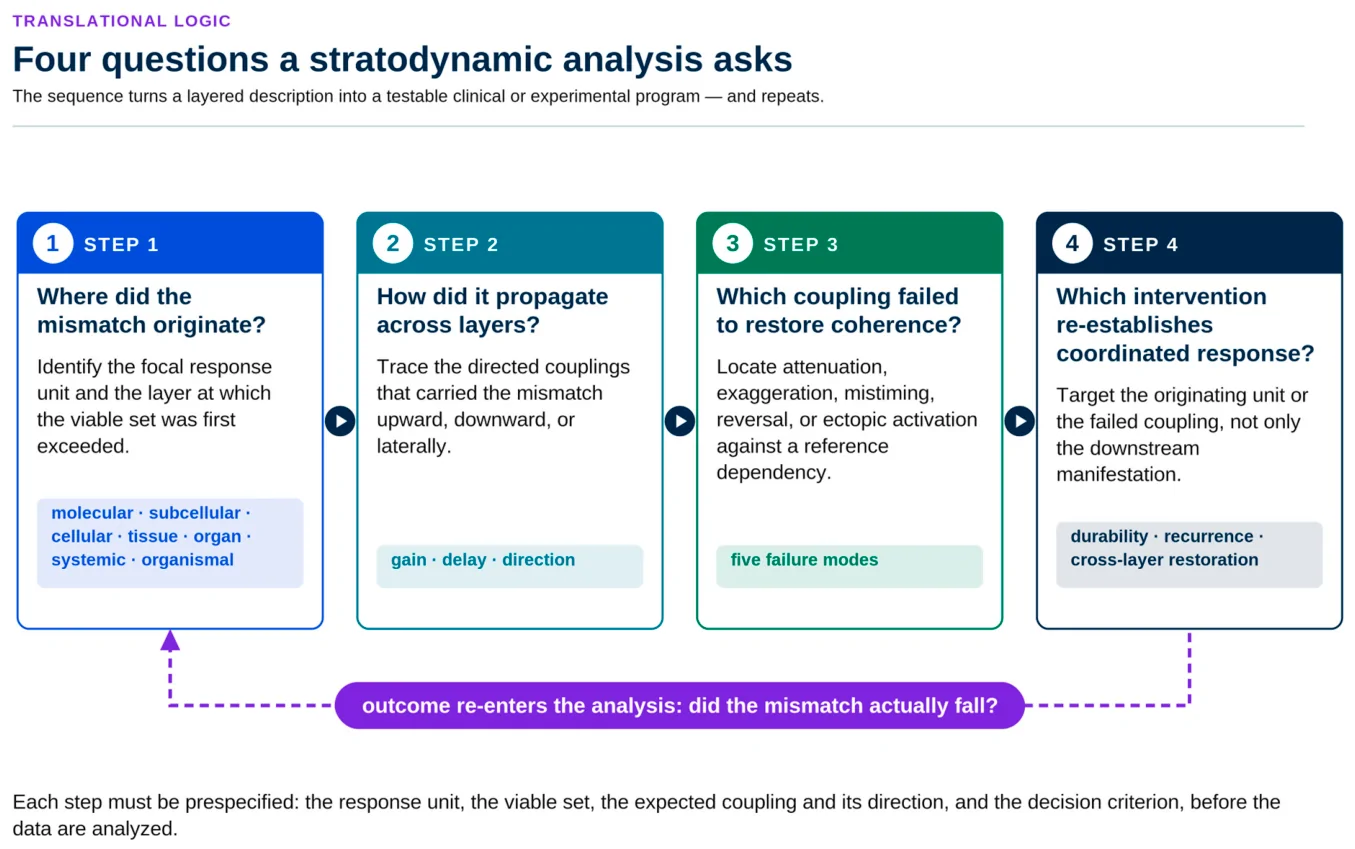
Translational logic of stratodynamics for veterinary and medical sciences. A stratodynamic approach asks where the mismatch originated, how it propagated across layers, which coupling failed to restore coherence, and which intervention can re-establish coordinated response. The framework may be especially useful for chronic inflammatory, metabolic, infectious, reproductive, degenerative, stress-related, and repair-associated diseases in vertebrates. Reading the figure: the four numbered panels are applied in sequence, each stating one question and the information required to answer it, with the chip beneath naming the quantities to be specified. The dashed return path indicates that the outcome re-enters the analysis as a test of whether the independently defined mismatch actually decreased, making the workflow a cycle rather than a linear checklist. Every element must be prespecified before analysis.

A stratodynamic analysis of disease or adaptive challenge should move beyond asking which molecule, cell type, organ, or systemic marker is abnormal. It should ask four linked questions: where did the mismatch originate; how did it propagate across layers; which coupling failed to restore coherence; and which intervention can re-establish coordinated response? The visible lesion or clinical sign may be the final expression of a longer chain rather than its origin. A hoof lesion, mammary inflammation, non-healing wound, metabolic disorder, or reproductive failure may appear local while its persistence depends on systemic inflammation, endocrine state, organ output, cellular phenotype, tissue repair capacity, and organismal behavior. Investigators should therefore distinguish initiating mismatch, propagating mechanisms, and failed resolution, and should define outcomes that demonstrate durable restoration rather than temporary suppression of one downstream marker. Figure 7 summarizes this translational workflow.

## 9. Limitations and Future Work

Stratodynamics is presented here as a new theory of layered biological response. It is an architectural framework intended to organize a research program, not a completed mathematical theory. Several limitations should therefore be stated plainly.

First, the framework is qualitative. The seven layers, their mismatch types, and their couplings are defined as biological and architectural relationships rather than as unified mathematical quantities. Each stratum can be analyzed with established tools: molecular thermodynamics at Layer 1; nonlinear signaling models and systems-biology methods at Layer 2; attractor dynamics and gene-regulatory networks at Layer 3; tissue mechanics, morphogen theory, bioelectricity, and resolution biology at Layer 4; organ physiology at Layer 5; cybernetic feedback and homeostasis at Layer 6; and allostasis, prediction, and active-inference models at Layer 7. These are analytical resources, not foundations of Stratodynamics. The mathematical challenge is to connect them in multiscale models of upward, downward, and lateral coupling. That work lies beyond the scope of the present paper. Recent viability-space and pre-disease-transition theories provide especially useful starting points for formalization: the former suggests explicit viable regions and response trajectories, whereas the latter offers measurable early-warning signals for declining resilience [59,60].

Second, the empirical and clinical implications of stratodynamics are hypotheses generated by the framework, not established clinical rules. The framework predicts that chronic, multifactorial, relapsing, and poorly resolving diseases often persist because inter-layer coupling fails to restore coherence. This prediction is consistent with many observations from inflammation, metabolic disease, autoimmunity, wound healing, stress biology, and chronic organ dysfunction, but it has not yet been tested prospectively as a stratodynamic prediction. Clinical use would require layer-resolved diagnostic methods, longitudinal sampling, causal modeling, and interventional studies able to determine whether correcting a proposed originating layer or failed coupling outperforms downstream symptom suppression. Until such evidence exists, stratodynamics should be understood as a research framework, not as guidance for clinical practice.

Third, the precise number of layers should be held provisionally. The central claim is not that nature is divided into exactly seven compartments. The claim is that biological response is scale-specific and that distinct response architectures become experimentally visible at different levels of organization. The seven-layer scheme proposed here, molecular, subcellular, cellular, tissue, organ, systemic, and organismal, is a best current articulation for complex multicellular animals, especially vertebrates. Other domains may require modification. Plants, unicellular organisms, microbial collectives, embryos, colonies, and ecosystems may require six-layer, eight-layer, or supraorganismal extensions [94,95]. The number seven should therefore be read as a functional architecture, not as a rigid ontology. Because six- or eight-layer alternatives can satisfy the same criteria, the choice among them is treated as an empirical question rather than a matter of argument. Under Prediction 1, the partition that should be adopted is the one that predicts held-out responses better than complexity-matched rivals: at a contested boundary—for example, treating intracellular signaling and organellar regulation as one subcellular stratum or as two—a model permitted to differ across that boundary should outperform models that merge or split it, and the advantage should reproduce across independent systems. Evidence that would favor seven is therefore reproducible predictive gain at each of the six retained boundaries, together with failure of merged or further-split alternatives to recover that gain.

Fourth, applying stratodynamics experimentally faces practical obstacles that should be stated plainly. Layer-resolved study requires simultaneous measurement at scales that are conventionally assayed separately, and a sampling frequency appropriate to one stratum may be far too sparse or too dense for another. Viable sets must be specified independently of the response being studied, which is straightforward for well-characterized systemic variables but difficult at subcellular and tissue scales, where context-dependent normal ranges are often unknown. Perturbing one coupling without disturbing others is frequently impossible in vivo; mediator redundancy can mask a real dependency; and unmeasured shared drivers cannot be excluded by statistical conditioning alone. Longitudinal layer-resolved designs are costly, and in food-producing and companion animals both sampling access and ethical constraints limit invasive measurement. These are reasons to begin in tractable systems—thermoregulation, cutaneous repair, or the periparturient transition—in which several strata are already measurable, rather than reasons to defer testing.

**Translational corollary.** Where a causally implicated upstream response unit or dysfunctional coupling has been established, restoring that unit or coupling is expected to produce more durable functional benefit and lower recurrence than downstream correction alone, when interventions are designed to achieve comparable initial downstream correction under prespecified safety constraints.

These limitations define the future program. Stratodynamics requires formalization, empirical validation, clinical testing, and domain-specific extension. It must be translated from an architectural theory into measurable variables, causal models, experimental designs, and therapeutic hypotheses. The present paper states the principle. The work that follows must determine how far the principle reaches. The framework’s principal falsifiable predictions are summarized in Box 4.

Box 4 **Falsifiable predictions of stratodynamics.**

Each application must prespecify response units and layer assignments, observables, viable sets, perturbation, expected direction and time window, comparator model, smallest effect of interest, and decision criterion. Adequately powered equivalence can refute an instantiated proposition; statistical non-significance alone cannot.

In the predictions below, *X_i_*(t) denotes the current or projected state of focal response unit *i*, *M_i_*(t) denotes its mismatch relative to the independently specified viable set V*_i_*(t), and δ denotes a prespecified smallest biologically meaningful difference or equivalence margin. Additional subscripts identify the outcome or intervention to which that margin applies.

Several predictions overlap with control theory, allostasis, causal systems biology, and network physiology. The distinctive claim is their integration within one prespecified layered, mismatch-based, directionally coupled architecture.

**Prediction 1—Response architectures are layer-specific.** Stratodynamics predicts that, in held-out response units or biological systems and after accounting for model complexity, a model allowing response dynamics to differ according to the prespecified layer assignments will outperform both (i) a constrained model in which the sampled layers follow one common response architecture differing only in gain and timescale and (ii) a complexity-matched model using shuffled or biologically plausible alternative layer assignments. The layer-specific architectures—molecular-state transition, signaling-threshold crossing, cell-fate commitment, distributed tissue program, integrated organ output, systemic regulated-range defense, and organismal anticipation—are dominant, nonexclusive prototypes at each layer, not behaviors that every unit at that layer must exhibit. The instantiated layer-specificity proposition is refuted if either comparator is equivalent or superior within a prespecified margin, such that the proposed layer assignment adds no reproducible predictive information.

**Prediction 2—Mismatch reduction predicts functional recovery beyond response magnitude alone.** In designs that experimentally dissociate response magnitude from mismatch reduction, and using an independent functional endpoint that was not used to define the viable set—later function, survival, or performance—Stratodynamics predicts that greater reduction of independently defined mismatch adds reproducible predictive information beyond response amplitude. The prediction is refuted if an amplitude-only model is equivalent or superior within a prespecified margin and adding mismatch reduction provides no reproducible incremental prediction.

**Prediction 3—A prespecified inter-layer coupling is directed and causally perturbable.** For a single specified coupling—with its source unit, target unit, expected direction, biologically meaningful magnitude, and response window stated in advance—Stratodynamics predicts that a controlled perturbation verified to engage the source produces the specified directed change at the target, and that blocking the proposed pathway, where feasible, attenuates or abolishes the target effect by a prespecified magnitude. An adequately powered equivalence result showing no prespecified target response after verified source engagement refutes the specified source–target coupling under the tested conditions. Persistence of the target response after selective and verified pathway blockade refutes the proposed mediator, but not necessarily the broader coupling. Statistical conditioning alone cannot exclude every unmeasured shared driver.

**Prediction 4—Chronic disease carries a cross-layer incoherence signature that precedes conventional single-layer failure.** In chronic diseases for which coupling dysfunction is hypothesized a priori, stratodynamics predicts that a measurable departure from an independently specified reference dependency —attenuation, exaggeration, mistiming, reversal, or ectopic activation of the expected cross-layer relationship—emerges before prespecified conventional single-layer markers or overt clinical transition and improves prediction of progression. The cross-layer model must be compared with a complexity-matched model that uses the same variables without cross-layer dependency terms. The instantiated prediction is refuted if either the cross-layer departure does not precede the prespecified single-layer marker or clinical transition by the stated lead time, or the complexity-matched model is equivalent or superior in out-of-sample prediction, such that the cross-layer dependency terms add no predictive information.

**Prediction 5—Predictive cues initiate responses before a current-state mismatch emerges.** With the current state of focal response unit *i*, *X_i_*(t), and its current mismatch, *M_i_*(t), held comparable, a cue that reliably predicts a future challenge should elicit a pre-challenge systemic or organismal response whose magnitude or timing varies with the cue’s predictive validity—that is, the strength and reliability of the experimentally established cue–challenge relationship. Altering that relationship should correspondingly modify the anticipatory response. The decisive comparators are an equally salient but nonpredictive cue and a purely reactive model driven only by the current distance of *X_i_*(t) from its viable set, V*_i_*(t). The instantiated prediction is refuted if, despite verified perception of the cue and successful manipulation of its predictive validity, either: (i) no response occurs before an actual mismatch develops; or (ii) prechallenge response magnitude and timing remain equivalent between predictive and nonpredictive cues and fail to update when the cue–challenge relationship changes. Here, equivalence means that any observed difference falls within prespecified bounds judged too small to be biologically meaningful. At the model level, equivalence or superiority of a current-mismatch-only model would refute the proposed anticipatory contribution. This prediction distinguishes stratodynamics from a purely reactive mismatch-correction model, although not from allostasis or other predictive-regulation theories.

**Illustrative instantiation A—Cellular-to-tissue coupling in wound repair.** In a standardized excisional-wound experiment, the prespecified source response could be a macrophage transition index defined from an independently selected marker panel, and the target response could be the re-epithelialization trajectory, including the rate of wound closure and time to barrier recovery. Source engagement would mean verified induction of the prespecified macrophage transition. Initial wound size, the primary response window, the smallest biologically meaningful difference in the target wound-healing trajectory, *δ_W_*, and the minimum reduction in the target effect expected after selective mediator blockade, *δ_B_*, would be defined before analysis from assay precision, prior evidence, and biological relevance.

The specified coupling would be refuted under the tested conditions if, after verified source engagement, the target trajectories were equivalent within the prespecified margin ±*δ_W_*, indicating no biologically meaningful target response. The proposed mediator would be refuted if selective blockade reduced the target effect by less than *δ_B_*, while source engagement remained intact. Failure of the mediator test would not necessarily refute the broader source–target coupling because another pathway could transmit the effect.

**Illustrative instantiation B—prospective transition-cow prediction.** In a cohort sampled from late gestation through 21 days in milk, a cross-layer model could prespecify lagged dependencies among organismal feed-intake or rumination behavior, systemic ionized-calcium and acute-phase responses, and mammary organ output. The comparator would contain the same variables and complexity but omit the cross-layer dependency terms. The minimum acceptable lead time and improvement in out-of-sample discrimination would be defined before model fitting. The instantiated prediction would be refuted if the dependency signature neither preceded the clinical or functional transition by the stated interval nor improved prediction beyond the complexity-matched comparator.

## 10. Conclusions

Claude Bernard recognized that higher organisms live not directly in the external world, but through an internal environment whose relative constancy makes free and independent life possible [1,27]. That recognition has organized physiology for more than a century and a half. It identified the central problem of biological regulation: how can a living organism preserve internal viability while moving through an external world that changes continuously?

Stratodynamics offers a synthesis of this lineage. It does not displace the predecessor frameworks. It locates them. Each framework is preserved at the scale where it is most precise and placed within a broader layered architecture of biological response. The principal recent frameworks are treated in the same way: as complementary accounts of network physiology, multiscale competency, whole-body function, viability, critical transition, resilience, and predictive self-maintenance that sharpen, but do not duplicate, the proposed response-centered seven-stratum architecture.

The central proposition of stratodynamics is that vertebrate biological response can be organized provisionally into seven functional response layers: molecular, subcellular, cellular, tissue, organ, systemic, and organismal. The framework predicts that each addresses a distinguishable form of mismatch through a characteristic but nonexclusive architecture. Whether this partition provides greater explanatory and predictive value than simpler, shuffled, or alternative partitions is an empirical question defined in Box 4.

The proposed unifying principle is mismatch reduction. Stratodynamics hypothesizes that successful biological responses reduce the distance between an actual or projected state and an independently specified viable set, while recognizing that the relevant state, viable range, and response architecture differ across scales. This proposition does not imply that every response succeeds, that a unique optimum exists, or that reducing mismatch at one layer necessarily reduces mismatch at every other layer.

The framework further proposes that these response layers are coupled upward, downward, and laterally. Upward coupling concerns the contribution of lower-layer activity to higher-layer integration; downward coupling concerns higher-layer context or constraint of lower-layer dynamics; and lateral coupling coordinates units operating at comparable scales. These classifications are hypotheses about directed dependencies, not evidence of causation by themselves. Their validity must be established through temporally resolved measurement, controlled perturbation where feasible, and comparison with alternative causal models.

Within this interpretation, Bernard’s milieu intérieur is maintained not by one mechanism but by the coordinated contribution of molecular transitions, intracellular signaling, cellular-state decisions, tissue programs, organ outputs, systemic feedback, and organismal anticipation. Stratodynamics proposes that the observed constancy of the internal environment depends on this layered coordination. Demonstrating that claim will require showing that prespecified cross-layer models explain or predict response better than complexity-matched alternatives lacking the proposed architecture.

For veterinary medicine, the framework generates the hypothesis that production, reproduction, immunity, locomotion, pain, behavior, metabolism, and tissue repair are coupled expressions of whole-animal response. The clustering of mastitis, lameness, metritis, ketosis, hypocalcemia, chronic inflammation, and impaired fertility is compatible with disturbed layered coherence, but does not establish it. Prospective layer-resolved studies must determine whether coupling measures improve prediction or intervention beyond established disease-specific variables.

For human medicine, the corresponding proposal is that some chronic inflammatory, metabolic, autoimmune, degenerative, septic-recovery, and repair-associated conditions may involve loss of coordination among molecular, cellular, tissue, organ, systemic, and organismal responses. Stratodynamics does not claim that such diseases have a single cross-layer cause or that layered analysis will necessarily outperform existing models. It supplies a structured way to test where mismatch originated, how it propagated, and whether restoration of a causally implicated coupling improves durable function.

The framework can therefore be summarized as a testable theoretical proposition:

Life persists not by maintaining constancy at one scale, but by actively reducing mismatch at every scale through layer-appropriate architectures coupled into one coherent response.

The paper closes where it began, with Bernard’s recognition that the relative constancy of the internal environment permits free and independent life. Stratodynamics proposes that this constancy may be better understood as layered coherence: not immobility of one internal condition, but coordinated response across organizational scales and through time. The present article defines that proposition and its principal empirical commitments; prospective experiments and comparative models must determine whether the proposed architecture is valid, where it fails, and how it should be revised.

## Figures and Tables

**Table 1:** Stratodynamics in relation to established and recent frameworks that overlap with its novelty claim. The five comparators retained here are those whose claims bear most directly on the novelty of stratodynamics; an extended comparison covering systems biology, multi-scale physiology, network medicine, five-domain physiology, cellular viability space, pre-disease criticality, and the free-energy principle is provided as Supplementary Table S1.

Framework	Primary Focus	Unifying Principle	Treatment of Coupling	Model of Disease	What Stratodynamics Adds
Homeostasis (Cannon) [4,5,6]	Defended variables, especially at systemic scale	Feedback regulation	Explicit in control loops; can span scales	Regulatory failure or displacement	Embeds regulated-range defense within a mismatch-based cross-layer architecture
Allostasis (Sterling, McEwen) [15,16,29]	Predictive, context-dependent organismal regulation	Stability through anticipatory change	Brain–body, endocrine, and autonomic coordination	Allostatic load and maladaptive prediction	Treats anticipation as a dominant organismal-scale architecture coupled to lower-layer responses
Hierarchy theory (Simon, Salthe, Noble) [44,45,52]	Nested composition, scale, constraints, near-decomposability, and multilevel causation	Varies among theories; no single accepted account of biological levels	Part–whole relations, boundary conditions, constraints, or cross-level causation	Not primarily a disease theory	Uses a limited response-specific compositional ordering without adopting emergence or no-privileged-level causation; adds stratum criteria, viable-set mismatch, and perturbable dependencies
Network physiology and adaptive physiological networks (Balagué et al.; Schöll et al.) [53,54]	Dynamic coordination among physiological and organ systems	Time-varying coordination among physiological systems	Explicit, dynamic, and potentially adaptive across systems and scales	Reorganization or breakdown of physiological-network interactions	Extends coupling across seven prespecified molecular-to-organismal response strata, with layer-specific architectures and viable-set mismatch
Multiscale competency (McMillen; Levin) [55,56,57]	Nested biological collectives and scale-appropriate problem solving	Goal-directed competency across levels	Collective signaling and multiscale coordination	Failure or misalignment of collective competency	Specifies seven vertebrate response strata, their architectures, and loss of cross-layer coherence

## Data Availability

No new data were created or analyzed in this theoretical manuscript. Data sharing is not applicable to this article.

## Supplementary Materials

The following supporting information can be downloaded at: https://www.mdpi.com/article/10.3390/biology15161426/s1, Table S1: Extended comparison of Stratodynamics with additional multi-scale and systems frameworks.
