# Supplementary material for: Stratodynamics: A New Theory of Layered Biological Response
Source: Biology (Basel). 2026 Aug 18;15(16):1426. doi: 10.3390/biology15161426 (PMC13509897; doi:10.3390/biology15161426)
Supplement: Supplementary file 1 [file biology-15-01426-s001.zip › biology-4498474-supplementary.pdf]

# Supplementary Material

*Stratodynamics: A New Theory of Layered Biological Response*

Burim N. Ametaj

**Table S1.** Extended comparison of Stratodynamics with additional multi-scale and systems frameworks. Table 1 in the main text retains the five comparators whose claims bear most directly on the novelty of Stratodynamics. The frameworks below overlap with the framework in scope or method and are reported here for completeness. Column headings follow Table 1.

| Framework | Primary focus | Unifying principle | Treatment of coupling | Model of disease | What stratodynamics adds |
|---|---|---|---|---|---|
| Systems biology | Quantitative analysis of interacting biological components and networks | System behavior from network structure and dynamics; some formulations emphasize non-additivity or emergence | Feedback, feed-forward, nonlinear, and cross-scale interactions | Network or state-space perturbation | Not derived from systems biology and does not require emergence, non-additivity, robustness, or network topology; adds seven response strata, viable-set mismatch, and directional dependency tests |
| Multi-scale physiology | Integration across spatial and temporal scales | Cross-scale model integration | Explicitly modeled across selected scales | Multiscale dysfunction | Adds a shared mismatch logic, layer-specific architectures, and refutable coupling propositions |
| Network medicine | Disease networks and modules | Network topology and disease-module organization | Network edges and intermodule relationships | Perturbation of disease modules and networks | Emphasizes directional coupling and coherence among organizational scales |
| Five-domain physiology [58] | Operational domains of whole-body function | Coherent integration of physiological functions | Cross-domain whole-body coordination | Dysfunction within or among functional domains | Adds a compositional seven-stratum response architecture and directional, perturbable couplings |
| Cellular viability space [59] | Cell-state trajectories relative to viable regions | Persistence within, or return toward, viability space | State-space relations among cellular variables | Exit from a viable region or irreversible death trajectory | Extends viable-set mismatch from the cellular scale across molecular-to-organismal response strata |
| Pre-disease criticality [60] | The tipping region between health and established disease | Declining resilience and early-warning dynamics | Network and multiscale transition signatures | Critical transition into a disease state | Locates transition dynamics within an origin–propagation–coupling account across specified response strata |
| Free-energy principle/active inference [34] | Self-maintenance through prediction and action | Limitation of variational free energy or prediction error | Organism–environment and multiscale inferential coupling | Maladaptive inference or failure of adaptive self-maintenance | Specifies the internal biological strata and response architectures through which self-maintenance may be implemented |
